# Molecular Characterization of Lasmiditan Binding to Human Serum Albumin: Site Preference, Structural Response, and Binding Energetics

**DOI:** 10.1021/acsomega.6c08646

**Published:** 2026-09-30

**Authors:** Tadeusz W. Inglot

**Affiliations:** Department of Medicinal Chemistry, Faculty of Pharmacy, Medical University of Lublin, Jaczewskiego 4, 20-090 Lublin, Poland

## Abstract

Human serum albumin (HSA), the major transport protein in blood plasma, can substantially influence the distribution of therapeutic compounds. Although lasmiditan (LASMI) is a recently introduced antimigraine drug with reported moderate plasma protein binding, the molecular determinants of its association with HSA remain poorly defined. Here, an integrated spectroscopic and computational approach was used to characterize HSA-LASMI complex formation, binding-site preference, and the accompanying structural response of the protein. UV–Vis absorption, steady-state fluorescence quenching, FTIR, and circular dichroism spectroscopy were combined with site-marker displacement experiments, molecular docking, 100 ns molecular dynamics simulations, and MM/PBSA binding free-energy calculations. Competitive site-marker experiments indicated preferential LASMI binding within Sudlow site I in subdomain IIA. Complex formation was associated with quenching of intrinsic HSA fluorescence and modest protein structural reorganization. Far-UV CD measurements showed a subtle LASMI concentration-dependent perturbation of the HSA secondary-structure-sensitive signal, with an apparent decrease in α-helical content from 53.16 ± 0.05 to 50.73 ± 0.15% at 96 μM LASMI. Molecular dynamics simulations further supported stabilization of the HSA-LASMI complex, as reflected by lower backbone RMSD values and reduced residue flexibility relative to unbound HSA. Thermodynamic analysis indicated spontaneous and favorable binding (e.g., Δ*G*_298 K_ = −21.76 kJ mol^–1^), while MM/PBSA calculations independently supported the energetic stability of the modeled complex. Together, these findings provide a molecular framework for understanding lasmiditan association with HSA and its potential relevance to plasma distribution and competition with co-administered Sudlow site I-binding compounds.

## Graphical abstract

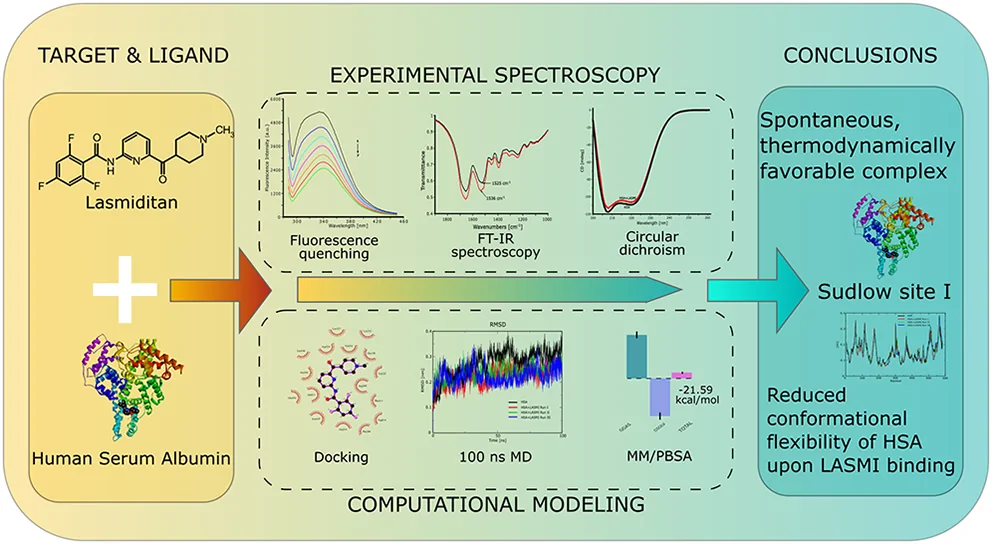

## 1 Introduction

Migraine represents a complex neurovascular disorder affecting approximately 15% of the global population, characterized by recurrent episodes of moderate to severe headache accompanied by neurological and gastrointestinal manifestations.^1,2^ The pathophysiology involves activation of the trigeminovascular system, resulting in the release of vasoactive neuropeptides, particularly calcitonin gene-related peptide (CGRP), which mediates vasodilation, inflammation, and nociceptive transmission.^2–5^ Contemporary understanding positions migraine as a primary neuronal sensory processing disorder with genetic predisposition to neuronal hyperexcitability, reducing the threshold for allostatic load on hypothalamic and trigeminal centers to generate paroxysmal cranial pain.^4,6^

Current pharmacological interventions encompass acute and preventive therapeutic strategies. Acute management historically relied on ergotamine derivatives and subsequently evolved to incorporate triptans, which function as serotonin 5-HT_1B/1D_ receptor agonists. However, these agents exhibit significant vasoconstrictive properties, precluding their use in patients with cardiovascular comorbidities.^4,6–11^ Preventive therapies predominantly utilize repurposed medications including antihypertensives, antidepressants, and antiepileptics, which demonstrate limited specificity for migraine pathophysiology.^2^

The identification of selective 5-HT_1F_ receptor agonism as an anti-nociceptive mechanism within the trigeminovascular system, devoid of vasoconstrictive effects, represented a significant therapeutic advancement.^3,4^ This discovery facilitated the development of ditans, a novel drug class targeting central neuronal mechanisms without peripheral vascular effects.^2,5,10^

Lasmiditan (2,4,6-trifluoro-N-[6-(1-methylpiperidine-4-carbonyl)­pyridin-2-yl]­benzamide) constitutes the first clinically approved ditan, exhibiting a unique pyridinoyl-piperidine scaffold that distinguishes it from indole-containing triptans (Figure 1). The compound demonstrates exceptional selectivity for human 5-HT_1F_ receptors (*K_i_* = 2.21 nM) compared to 5-HT_1A/B/D_ receptors (*K_i_* > 1000 nM), representing greater than 440-fold selectivity over vasoconstrictive receptor subtypes.^12,13^ This selectivity profile eliminates discernible agonist activity at 5-HT_1B/D_ receptors while maintaining nanomolar efficacy at 5-HT_1F_ receptors.^11^

**Figure 1:**
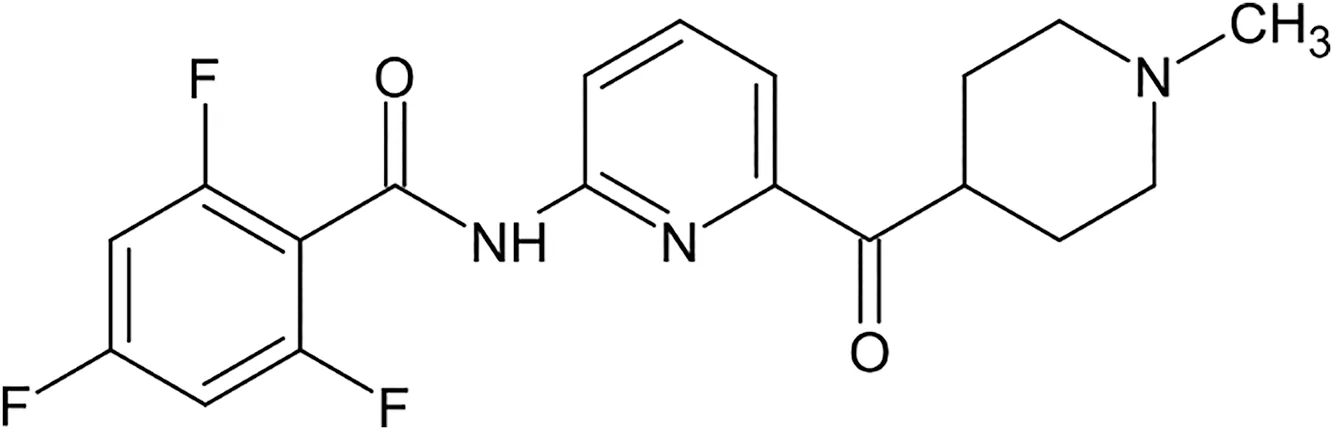
Chemical structure of lasmiditan (LASMI).

The proposed mechanism of action involves presynaptic binding at peripheral trigeminovascular nerve terminals, resulting in reduced CGRP release, while central actions at trigeminal, thalamic, and cortical sites diminish glutamatergic tone through stimulation of inhibitory 5-HT_1F_ receptors on glutamatergic neurons implicated in central sensitization.^4,11,13^ The compound’s lipophilic nature enables blood-brain barrier penetration, distinguishing it from peripherally restricted triptans and contributing to its central nervous system effects.^7,14^

Pharmacokinetic analysis reveals rapid oral absorption with median *T*_max_ of 1.8 h, moderate protein binding (55–60%), and elimination half-life of 5.7 h.^4^ High-fat meal co-administration increases *C*_max_ and AUC by 22 and 19% respectively, with 1 h *T*_max_ delay, though clinical significance remains minimal. Metabolism occurs primarily through non-cytochrome P450 pathways via ketone reduction, generating inactive metabolites M7 and M18, with minimal renal excretion (3% unchanged drug).^13^ The major urinary metabolite S-M8 represents 66% of administered dose, with recovery predominantly within 48 h.^4^

Clinical efficacy demonstrated superiority over placebo in phase III trials, with headache freedom rates of 28.2 and 32.2% for 100 mg and 200 mg doses respectively at 2 h post-administration. Adverse event profiles included dose-dependent central nervous system effects, primarily dizziness (12.5–16.3%) and fatigue, with onset occurring 30–50 min post-administration and duration of 1.5–2 h.^10^ Notably, cardiovascular safety analysis in patients with multiple risk factors revealed no significant cardiovascular adverse events compared to placebo.^1,3^

Human serum albumin (HSA) is the predominant plasma protein, playing a vital role in transporting various endogenous biomolecules and metabolites across the circulatory system. This monomeric, globular polypeptide of 585 amino acids adopts a spatial structure composed of three homologous domains (I–III), each subdivided into A and B subdomains.^15^ It presents multiple ligand-binding pockets: Sudlow’s Site I in subdomain IIA, Sudlow’s Site II in subdomain IIIA, and an auxiliary Site III located in subdomain IB, which together confer broad molecular recognition capabilities.^16–18^ Such a multisite architecture underlies HSA’s function as a carrier for numerous pharmacological agents. In particular, HSA can increase the aqueous solubility of hydrophobic drugs by forming reversible complexes in plasma, where non-covalent interactions (hydrogen bonds, hydrophobic contacts, electrostatic attractions and van der Waals forces) stabilize the binding and facilitate efficient distribution to target tissues.^19,20^ In addition to conventional small-molecule organic therapeutics, human serum albumin has increasingly been explored as an endogenous carrier and targeted delivery platform for diverse pharmacological scaffolds, including functionalized transition-metal complexes tailored for specific binding subdomains and active residues.^21,22^

Available pharmacokinetic information on lasmiditan (LASMI) binding to unspecified plasma proteins is limited to the REYVOW prescribing information, which indicates that 55–60% of the compound is protein-bound in blood.^23,24^ Given the lipophilic nature of lasmiditan and its moderate plasma protein binding, characterizing the biophysical affinity and thermodynamic parameters of the HSA-LASMI interaction is important for understanding the unbound drug fraction. Because albumin binding may influence the plasma distribution of lasmiditan, it may also be relevant to its potential central nervous system exposure. In the absence of detailed biophysical studies of HSA-LASMI association, the present work systematically investigates this interaction using UV–Vis absorption, circular dichroism, Fourier-transform infrared (FT-IR), and fluorescence spectroscopy, including steady-state, synchronous, and three-dimensional excitation-emission matrix measurements, under conditions mimicking physiological pH (7.4). To enrich the experimental data, computational strategies are also applied: molecular docking to predict binding poses within HSA’s known ligand sites, followed by molecular dynamics simulations to probe the stability and conformational dynamics of the HSA-LASMI complexes at the atomic level. To the best of my knowledge, this is the first study to combine multi-spectroscopic characterization, competitive site-marker analysis, and replicated molecular dynamics simulations to define the molecular features of lasmiditan binding to HSA.

## 2 Materials and Methods

### 2.1 Reagents

Human serum albumin (fatty acid- and globulin-free, ≥99% purity), lidocaine HCl, and ibuprofen were procured from Sigma-Aldrich/Merck (USA). TargetMol (USA) supplied both lasmiditan and warfarin sodium. A 10× concentrated phosphate-buffered saline (PBS) stock solution was obtained from Fisher Scientific (Germany). All aqueous solutions were prepared using ultrapure deionized water (conductivity <0.05 μS/cm) produced by a SolPure 7 apparatus (Alchem, Poland).

### 2.2 Instrumentation

Steady-state, synchronous, and three-dimensional fluorescence spectra were acquired using a Shimadzu RF-6000 spectrofluorometer (Japan) equipped with a Peltier-type temperature controller. UV–Vis absorbance measurements were executed on a Hitachi U-2001 spectrophotometer (Japan), whereas circular dichroism (CD) profiles were recorded with a Jasco J-815 spectropolarimeter (Japan) featuring a thermostated cell holder. Fourier-transform infrared (FT-IR) spectra were collected on a Nicolet 6700 spectrometer (Thermo Scientific, USA). Solution pH was monitored using a freshly calibrated Hanna Instruments HI9125 pH meter (USA). All optical analyses utilized high-purity quartz cuvettes: a 2 mm path length was employed for CD measurements, while standard 10 mm path-length cuvettes were used for all remaining spectroscopic techniques.

### 2.3 Sample Preparation

All experimental solutions were formulated using a phosphate-buffered saline (PBS) working buffer, prepared via a 10-fold dilution of the concentrated stock; the final pH of 7.4 was verified using a calibrated pH meter. A 20 μM HSA stock solution was formulated directly in PBS. Stock solutions of lasmiditan (4 mM) and ibuprofen (4 mM) required initial dissolution in 1 mL of methanol prior to volume adjustment with PBS. Meanwhile, 4 mM solutions of lidocaine hydrochloride and warfarin sodium were dissolved directly in pure PBS. All prepared solutions remained chemically and physically stable throughout the experimental timeframe and were stored at 4 °C protected from light. The maximum final methanol concentration used as a co-solvent was 0.48% (v/v). Control experiments confirmed that this methanol concentration did not cause significant changes in the UV–Vis absorption, far-UV CD, or fluorescence spectra of HSA.

### 2.4 UV–Vis Absorption Measurements

HSA-LASMI complexes were formulated by adding increasing volumes (12 μL increments) of LASMI to separate 5 mL flasks containing 4 μM HSA, resulting in final ligand concentrations ranging from 0 to 96 μM. After a 30 min incubation at room temperature, absorbance spectra were collected from 240 to 400 nm relative to a PBS reference. To compensate for the intrinsic UV absorption of LASMI below 280 nm, control spectra of identical ligand concentrations in protein-free PBS were recorded and mathematically subtracted from the raw mixture spectra. The entire procedure was performed in triplicate (*n* = 3).

### 2.5 Steady-State Fluorescence Spectroscopy

A 4 μM HSA solution in PBS (pH 7.4) was distributed into separate 5 mL volumetric flasks and treated with varying aliquots of LASMI to achieve a ligand concentration series ranging from 0 to 96 μM. The mixtures were incubated in the dark at 25 °C for 30 min. Subsequently, samples were transferred into a four-sided polished quartz cuvette and allowed to thermally equilibrate inside the spectrofluorometer for 10 min following each temperature adjustment. Emission spectra were recorded from 290 to 450 nm using an excitation wavelength of 280 nm. Both excitation and emission slit widths were set to 5 nm, with a scanning speed of 600 nm/min. To determine the thermodynamic parameters of the protein-ligand association, quenching experiments were conducted at three temperatures (298.15, 308.15, and 318.15 K). Appropriate buffer background (PBS) was subtracted from all experimental spectra. Furthermore, because LASMI exhibits noticeable UV absorption at 280 nm (with negligible absorption at 340 nm), the observed fluorescence intensities were corrected for the inner-filter effect (IFE) using the following equation:^25^

1 $$F_{corr}=F_{obs}\times 10^{\left(A_{ex}+A_{em}\right)/2}$$

where *F*_corr_ and *F*_obs_ denote the corrected and experimentally observed fluorescence intensities, respectively, while *A*_ex_ and *A*_em_ represent the absorbances of the system at the excitation (280 nm) and emission (340 nm) wavelengths. The entire multi-temperature quenching protocol was performed in triplicate (*n* = 3) using freshly prepared solutions. Independent control scans confirmed that LASMI alone emitted no detectable fluorescence within the 290–450 nm range under the experimental conditions. The Stern–Volmer analysis was used to examine how fluorescence quenching changes with temperature, and the double-logarithmic analysis was used separately to estimate the apparent binding constant and the binding stoichiometry. The binding constants obtained at different temperatures from the double-logarithmic analysis were then used in the van’t Hoff analysis.

### 2.6 Synchronous and 3D EEM Fluorescence Spectroscopy

To evaluate structural perturbations around specific aromatic fluorophores, synchronous spectra of 4 μM HSA titrated with LASMI (9.6–96 μM) were collected at fixed wavelength differences of Δλ = 15 nm (tyrosine-selective) and Δλ = 60 nm (tryptophan-selective) under identical instrumental and incubation conditions (30 min at 25 °C) as described for steady-state fluorescence.

Three-dimensional excitation-emission matrices (3D EEM) for free HSA (4 μM) and the 96 μM LASMI complex were generated after a 30 min incubation at 25 °C. The scanning range encompassed λ_ex_ = 200–400 nm (5 nm steps) and λ_ex_ = 200–500 nm (2 nm steps) at a scan speed of 1200 nm/min.

### 2.7 Competitive Site Marker Binding Analysis

To identify the primary binding location of LASMI on human serum albumin, competitive displacement experiments were conducted using three well-established site probes: warfarin for Sudlow’s Site I, ibuprofen for Sudlow’s Site II,^26^ and lidocaine for Site III.^27^ HSA, maintained at a concentration of 4 μM in PBS, pH 7.4, was pre-incubated with warfarin, ibuprofen, or lidocaine at a final concentration of 4 μM, corresponding to a 1:1 molar ratio, for 30 min at 25 °C. LASMI was subsequently added to obtain final concentrations of 16, 32, 48, 64, 80, and 96 μM. Following an additional 30 min incubation at 25 °C in the dark, fluorescence emission spectra were acquired using excitation at 280 nm and emission scanning from 290 to 450 nm. The corresponding HSA-marker solutions without LASMI were measured under identical conditions and used as the relevant *F*_0_ controls for each titration series. Absorbance spectra were recorded for every sample at the excitation wavelength of 280 nm and at 340 nm and fluorescence intensities were corrected for the inner-filter effect. Each complete titration series was performed using three independently prepared samples (*n* = 3). Results are reported as mean ± SD.

### 2.8 Circular Dichroism Spectroscopy

Far-UV CD spectra of pure HSA (4 μM in PBS, pH 7.4) and the HSA-LASMI complex (at final ligand concentrations of 32, 64, and 96 μM) were recorded at 25 °C using a quartz cell with a 2 mm optical path length. Spectra were scanned in the 200–260 nm region at a continuous rate of 50 nm/min. The baseline spectrum of the pure PBS buffer was acquired under identical parameters and mathematically subtracted from each sample spectrum. Control analyses confirmed that free LASMI exhibited no differential absorption of left- and right-circularly polarized light within the evaluated wavelength range. Each concentration was analyzed using three independently prepared HSA-LASMI samples (*n* = 3). To optimize the signal-to-noise ratio, three consecutive scans were accumulated for each sample and automatically averaged by the instrument software. Mean spectra and quantitative CD parameters are reported as mean ± standard deviation (SD). Mean residue ellipticity at 222 nm, [θ]_222_, was calculated according to

2 $${\left[\theta \right]}_{222}=\frac{{CD}_{222}\times MRW}{10\mathrm{cl}}$$

where CD_222_ is the observed ellipticity in mdeg, MRW is the mean residue weight of HSA (113.57 Da), *c* is the protein concentration in mg mL^–1^, and *l* is the optical path length in cm. The apparent α-helical content was estimated using the Chen-Yang equation:^28^

3 $$\alpha helix\left(\%\right)=\frac{{-\left[\theta \right]}_{222}-2340}{30300}\times 100$$

### 2.9 Solid-State FT-IR Measurements

To examine ligand-induced changes in protein backbone vibrations, 400 μL of a 100 μM HSA solution prepared in deionized water (without buffer) was mixed with 10 μL of LASMI at a 1:1 molar ratio in an agate mortar. The mixture was equilibrated for 30 min at 25 °C and dried under a stream of nitrogen. The resulting solid residue was ground with 300 mg of anhydrous KBr and pressed into a pellet for transmission FT-IR analysis using a Nicolet 6700 spectrometer. Control pellets containing native HSA (400 μL) or LASMI alone (10 μL) were prepared using the same procedure. Spectra were acquired over the range of 4000–500 cm^–1^ at a resolution of 2 cm^–1^, with 32 scans averaged per spectrum.

### 2.10 Computational Docking Studies

Molecular docking was performed with AutoDock Vina 1.2.7 ^29^ implemented in PyRx 0.8.^30^ Two HSA crystal structures from the PDB were used: 1AO6 ^31^ for blind and site-specific docking of LASMI, and 2BXD (warfarin-bound)^32^ for protocol validation and for testing the effect of warfarin occupancy on LASMI docking. Chain A was used in all calculations. For receptor preparation, water molecules and non-protein atoms were removed from 1AO6. The same was done for 2BXD, except that warfarin was kept as a rigid part of the receptor in the warfarin-occupied docking calculation. Polar hydrogens were added and Gasteiger charges were assigned with PyRx. The LASMI structure was optimized at the B3LYP-D3BJ/6-311++G­(2d,p) level in ORCA 6.1.1 ^33^ before docking. The optimized structure was converted to PDBQT format with rotatable bonds defined. Blind docking of LASMI into 1AO6 used a 100 × 100 × 100 Å search box centered on the protein. Five independent runs were performed with exhaustiveness 32 and 9 output poses retained per run. Site-specific docking was performed for Sudlow Site I (centered on Trp214), Site II (Tyr411), and Site III (Arg186), each with a 24 × 36 × 24 Å search box. Five runs were carried out per site with the same settings. Grid-box centres are listed in Table S2. The docking protocol was validated by redocking the crystallographic warfarin from 2BXD back into Site I of the same structure (without warfarin). Five runs were performed, and the heavy-atom RMSD between the top-ranked redocked pose and the crystallographic warfarin was calculated after protein superposition. To test the effect of Site I occupancy, LASMI was also docked into 2BXD with warfarin kept as a rigid part of the receptor, using the same Site I grid and settings. This was done to check whether LASMI can still bind inside Site I when warfarin is present, or whether it is redirected to a different location. Complete docking scores are presented in Table S3. The pose used for MD simulation was not chosen only by the lowest Vina score. It was selected from the Site I poses that appeared repeatedly across independent runs, had no major steric clashes, and agreed with the Site I preference indicated by the fluorescence competition experiments. Two- and three-dimensional mapping of intermolecular interactions for the top-scoring binding poses was executed using LigPlot+^34^ and Discovery Studio 2024, respectively.

### 2.11 Molecular Dynamics Simulations

Molecular dynamics simulations were performed using GROMACS 2024.5.^35^ HSA was described using the CHARMM36 force field,^36^ as implemented in the CHARMM36 February 2026/CGenFF 5.0 force-field distribution. LASMI parameters were obtained from the CHARMM General Force Field (CGenFF);^37^ the ligand topology and parameter files were included together with the protein topology. The reported CGenFF penalty score for LASMI was 3.476. The simulations used the CHARMM-compatible TIP3P water model. The HSA-LASMI complex was placed in a dodecahedral periodic box with a minimum solute-to-box distance of 1.0 nm.^38^ The system contained one HSA molecule, one LASMI molecule, 31,424 water molecules, and 14 Na^+^ counterions for charge neutralization. Periodic boundary conditions were applied in all three dimensions. Energy minimization was carried out using the steepest-descent algorithm for a maximum of 50,000 steps. The Verlet cutoff scheme was used with a neighbour-list cutoff of 1.2 nm. Long-range electrostatic interactions were treated with the particle-mesh Ewald method using a real-space cutoff of 1.2 nm, fourth-order interpolation, and a Fourier grid spacing of 0.16 nm. No long-range dispersion correction was applied, in accordance with the CHARMM36 force-field settings. After minimization, the system was equilibrated in two stages. First, a 100 ps NVT equilibration was performed at 300 K. This was followed by a 100 ps NPT equilibration at 300 K and 1 bar. During both equilibration stages, position restraints were applied to the protein and ligand heavy atoms. Temperature was maintained using the V-rescale thermostat with separate coupling groups for protein-ligand and water-Na^+^ components, a coupling constant of 0.1 ps, and a reference temperature of 300 K. During NPT equilibration, pressure was controlled isotropically using the C-rescale barostat, with a coupling constant of 2.0 ps, and a reference pressure of 1 bar. Production simulations were subsequently performed without position restraints in the NPT ensemble for 100 ns using a leap-frog integrator and a 2 fs time step. All bonds involving hydrogen atoms were constrained using the LINCS algorithm (fourth-order expansion; one iteration). Temperature control was maintained with the V-rescale thermostat under the same coupling scheme as during equilibration. Pressure was controlled isotropically using the Parrinello–Rahman barostat with a coupling constant of 2.0 ps, and a reference pressure of 1 bar. Coordinates, energies, and log information were written every 10 ps. Three independent 100 ns simulations were performed for the HSA-LASMI complex, providing an aggregate production time of 300 ns. The unbound HSA system was simulated for 100 ns using the same production settings.

The binding free energy (Δ*G*_bind_) was calculated using the gmx_MMPBSA tool, which combines GROMACS trajectories with AMBER continuum solvation models.^39^ The total binding energy was determined by summing the gas-phase molecular mechanics energies (van der Waals and electrostatic interactions) and the solvation free energies calculated with the Poisson-Boltzmann (PB) model.

## 3 Results and Discussion

### 3.1 UV–Vis Spectrophotometric Analysis

To detect ground-state complexation and evaluate conformational changes in protein frameworks, UV–Vis absorption scanning provides a highly sensitive analytical tool. In human serum albumin, the prominent absorption peak at 280 nm is driven predominantly by π→π* electronic transitions localized within its aromatic chromophores, namely tryptophan, tyrosine, and phenylalanine.^40^

Because lasmiditan displays intrinsic absorption at wavelengths shorter than 280 nm, Figure 2 presents the absorption spectrum of native albumin alongside the corrected spectra of the protein-ligand complexes, obtained by mathematically subtracting the pure ligand spectra at equivalent concentrations. The apparent absorption maximum shifted from 279.5 nm for free HSA to 278.5 nm in the presence of LASMI, corresponding to a slight 1.0 nm hypsochromic shift that was observed in three independently prepared samples. The corrected absorbance of HSA decreased by 0.029 absorbance units (13.4%) as shown in Table S1. The concentration-dependent decrease was monotonic across the titration series. This behavior likely points to π–π stacking interactions between the aromatic rings of the protein residues and the pyridine and trifluorophenyl moieties of LASMI.^41^ The observed absorption changes support ground-state complex formation. They are also consistent with a static contribution to fluorescence quenching, as dynamic quenching affects only the excited state and typically does not alter the UV–Vis absorption profile.^42^

**Figure 2:**
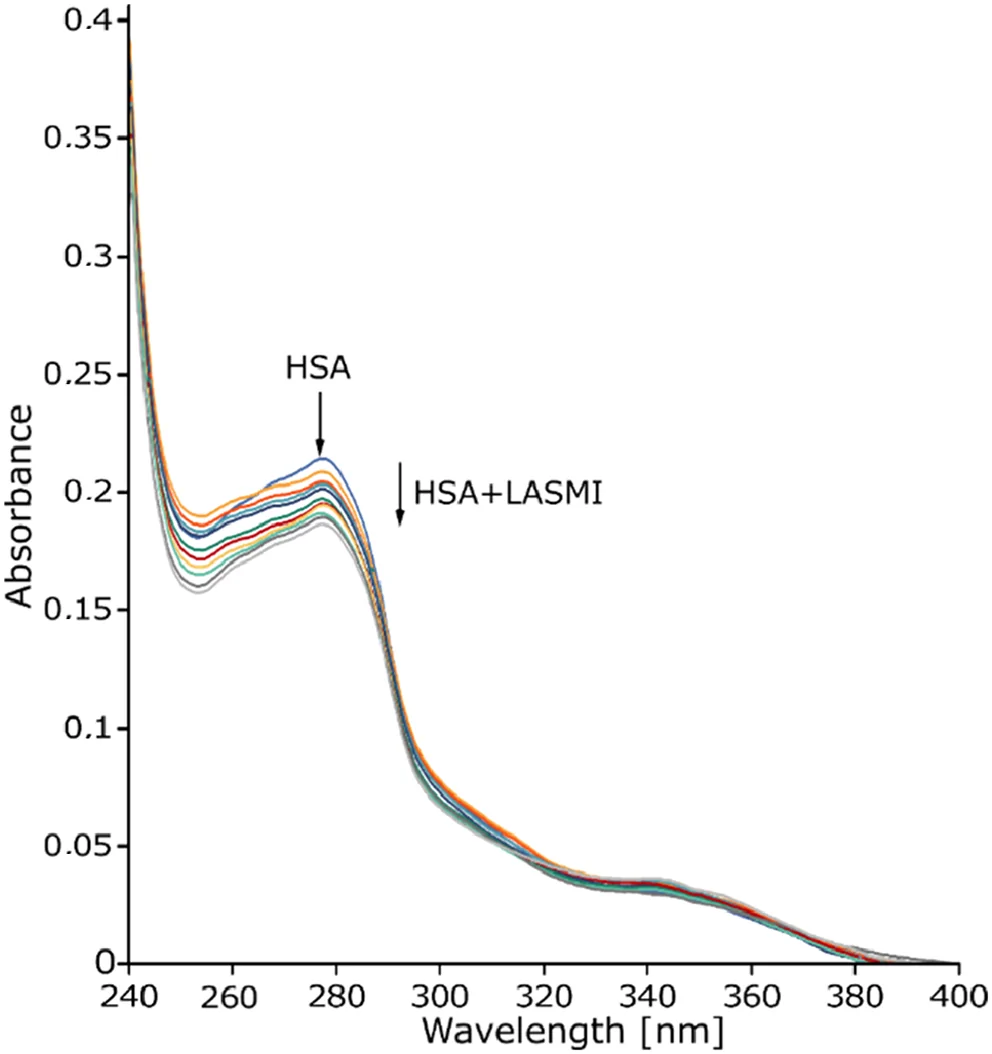
UV–Vis absorption spectra of free HSA (4 μM) and HSA complexes with LASMI (9.6–96 μM) obtained after subtracting the absorbance of pure LASMI at corresponding concentrations.

### 3.2 Fluorescence Quenching

Fluorescence spectroscopy represents a fundamental analytical method for investigating intermolecular interactions within protein–ligand systems. The intrinsic emission of proteins originates from aromatic amino acid residues: tryptophan (Trp), tyrosine (Tyr), and phenylalanine (Phe). In human serum albumin (HSA), the overall emission profile is dominated by Trp due to the exceptionally high quantum yield of its indole chromophore. The contribution of Tyr is noticeably lower, while the impact of Phe remains negligible due to its extremely poor emission efficiency, even though 31 such residues are distributed across the HSA polypeptide chain. Importantly, Trp emission is highly sensitive to fluctuations in its microenvironment. The binding of a ligand alters local polarity or introduces novel non-covalent interactions that induce structural rearrangements within the protein matrix. This perturbation results in a reduction of emission intensity, Such fluorescence quenching can proceed through static complexation in the ground state, dynamic collisional encounters, or non-radiative FRET mechanisms.^25,43,44^ Temperature-dependent quenching analysis provides a definitive means to distinguish between these pathways: static quenching constants decrease at higher temperatures due to enhanced thermal dissociation of the complex, whereas dynamic quenching constants increase as elevated temperatures accelerate diffusion-controlled molecular collisions.^45,46^

The quenching kinetics were mathematically modeled using the Stern–Volmer relationship:

4 $$\frac{F_{0}}{F}=1+K_{\mathrm{SV}}\left[Q\right]=1+k_{q}\tau_{0}\left[Q\right]$$

in which *F*_0_ and *F* denote the fluorescence intensities in the absence and presence of quencher concentration [*Q*], respectively. *K*_SV_ is the Stern–Volmer constant, and *k*_q_ is the bimolecular quenching rate constant assuming an uncomplexed albumin lifetime (τ_0_ of 10^–8^ s).^25^ At each temperature, the Stern–Volmer plot was made by plotting *F*_0_/*F* against the total LASMI concentration (Figure 4A). The straight line was fitted with the intercept fixed at 1.0, as required by the Stern–Volmer equation. The slope of this line gives *K*_SV_, and the quenching rate constant was calculated as *k*_q_ = *K*_SV_/τ_0_.

Figure 3 shows HSA emission profiles (λ_ex_ = 280 nm, 298 K) titrated with LASMI. While raw fluorescence drops sharply upon ligand addition, correction for the inner-filter effect (IFE), required by LASMI absorption at 280 nm, reveals moderate quenching constants (Table 1). Furthermore, titrations induce a bathochromic shift from 339 to 343 nm, indicating increased polarity in the Trp microenvironment caused by the insertion of polar ligand groups into the binding cavity.^47^ Control assays confirmed LASMI is non-fluorescent. The way *K*_SV_ changes with temperature gives information about the type of quenching. The *K*_SV_ values decreased from 1.29 × 10^3^ M^–1^ at 298 K to 1.03 × 10^3^ M^–1^ at 318 K. This is what is expected when a ground-state HSA-LASMI complex becomes less stable at higher temperature. Purely dynamic (collision-based) quenching would instead be expected to become stronger at higher temperature. In addition, the calculated *k*_q_ values were higher than the typical limit for diffusion in water (2.0 × 10^10^ L mol^–1^ s^–1^).^48^ Taken together with the changes seen in the UV–Vis absorption spectra of HSA after adding LASMI, these results indicate that quenching is mostly static. However, because fluorescence lifetimes were not measured, a small dynamic component cannot be ruled out.^49^

**Figure 3:**
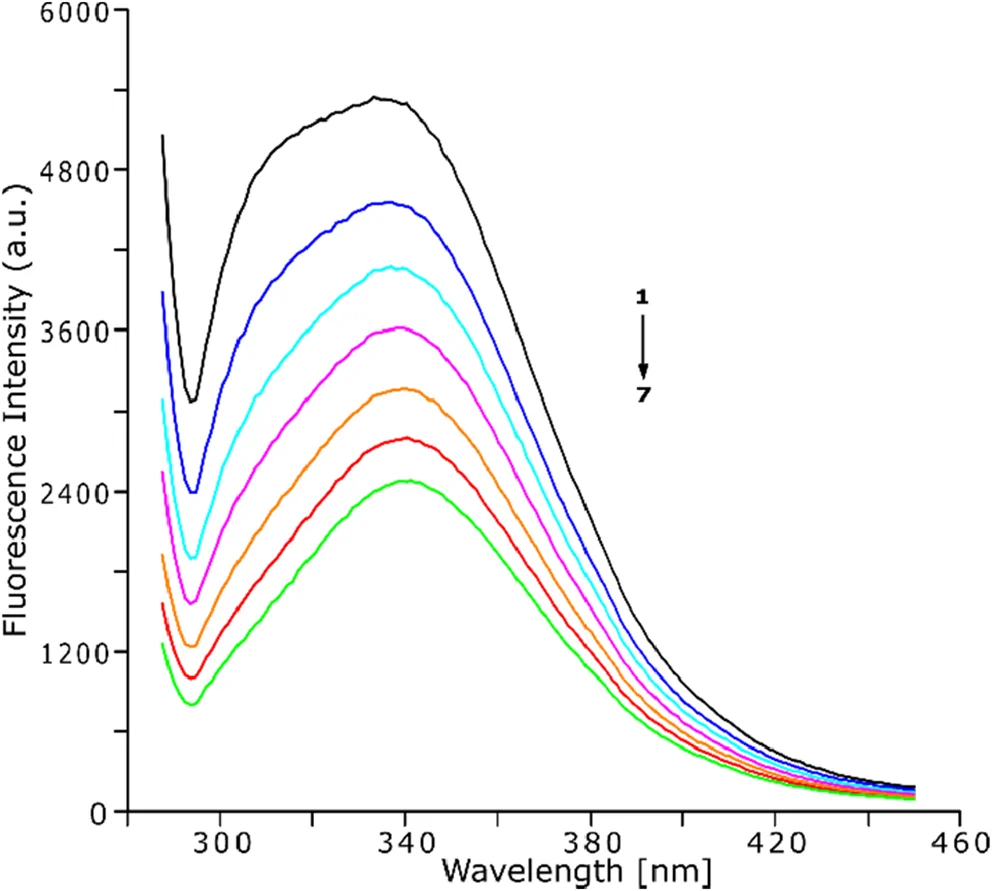
Fluorescence emission spectra of free HSA and HSA–LASMI complexes with increasing concentrations of LASMI (16, 32, 48, 64, 80, 96 μM) at 298 K (λ_ex_ = 280 nm).

**Table 1:** Stern–Volmer Quenching Constants (*K*_SV_), Bimolecular Quenching Rate Constants (*k*_q_), Association Constants (*K*_a_), and the Binding Stoichiometry (*n*) for the HSA-LASMI Interaction at 298, 308, and 318 K

*T* [K]	*K*_SV_ [×10^3^ M^–1^]	*k*_q_ [×10^11^ M^–1^s^–1^]	*K*_a_ [×10^3^ M^–1^]	*n*
298	1.29 ± 0.004	1.29 ± 0.004	6.46 ± 0.71	1.17 ± 0.01
308	1.15 ± 0.04	1.15 ± 0.04	3.70 ± 0.73	1.12 ± 0.03
318	1.03 ± 0.02	1.03 ± 0.02	2.04 ± 0.42	1.07 ± 0.02

### 3.3 Association Constant and Number of Binding Sites

To quantitatively determine the binding affinity and stoichiometry of the protein–ligand interaction, the association constant (*K*_a_) and the binding stoichiometry (*n*) were evaluated using the classical double-logarithmic equation:^50^

5 $$\log\left(\frac{F_{0}-F}{F}\right)=\log K_{a}+n\log\left[Q\right]$$

In this plot, the intercept gives log *K*_a_ and the slope gives the binding stoichiometry (*n*). The *K*_a_ values obtained in this way were used for the van’t Hoff analysis. High linear correlation coefficients (*R* > 0.99) confirmed the validity of eq 5 for this system (Figure 4B). The calculated value of *n* is approximately equal to 1, which suggests a 1:1 binding stoichiometry and implies the existence of a single primary binding site for LASMI on the HSA molecule. Furthermore, consistent with static quenching, *K*_a_ values decreased at higher temperatures, reflecting the thermal destabilization of the protein-ligand complex.

**Figure 4:**
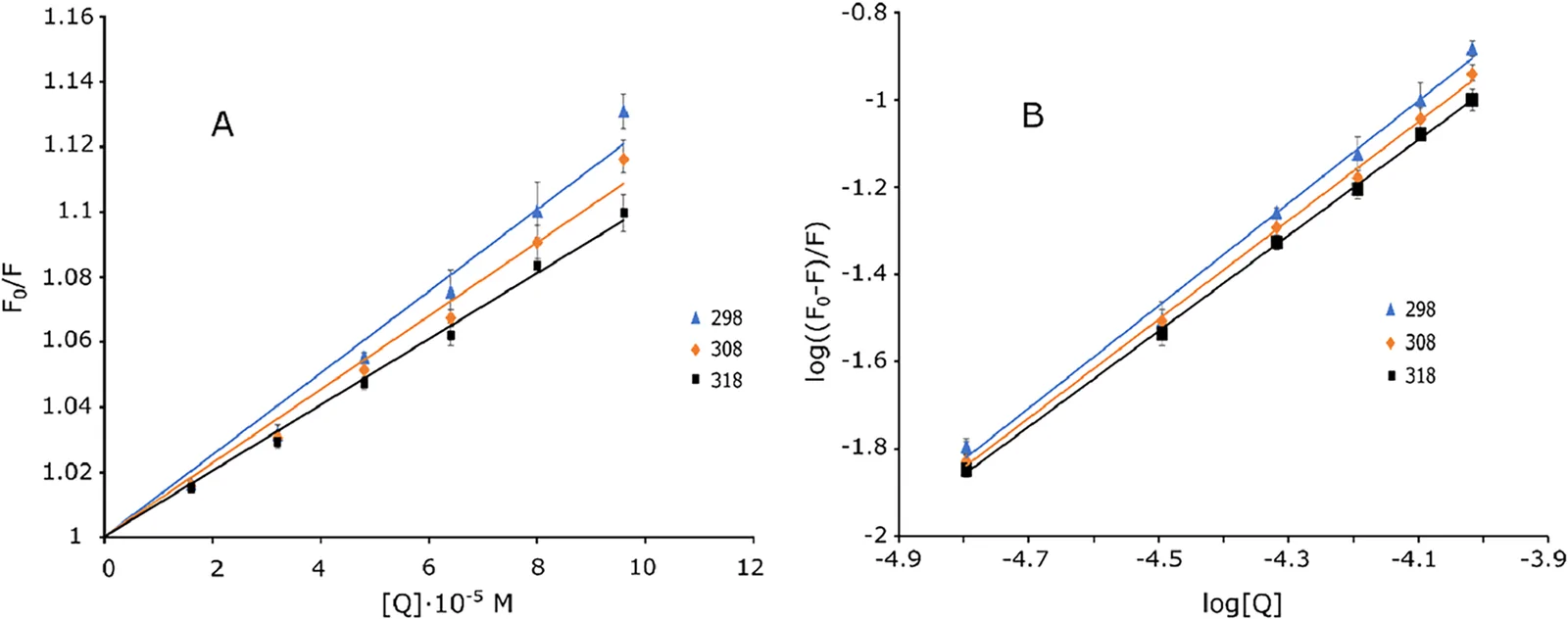
(A) Stern–Volmer plots for the quenching of HSA fluorescence by LASMI at 298, 308, and 318 K. (B) Double-logarithmic plots for determining the binding constant and the binding stoichiometry.

### 3.4 Synchronous Fluorescence Spectra

To investigate structural alterations around aromatic amino acid residues upon ligand binding, synchronous fluorescence spectroscopy was performed. Wavelength intervals of Δλ = 15 and 60 nm were applied to selectively monitor the spectroscopic behavior of tyrosine and tryptophan residues, respectively. However, the quenching observed at Δλ = 60 nm (Figure 5) was accompanied by a bathochromic shift of the emission maximum from 292 to 294 nm. This shift suggests that ligand complexation exposes the tryptophan residues to a more polar aqueous environment. Conversely, the emission band at Δλ = 15 nm exhibited no discernible wavelength shift. These combined observations indicate that LASMI probably perturbs the chemical microenvironment in the vicinity of the Trp residue while leaving the polarity surrounding the tyrosine residues largely unaffected.^51^ Given the lack of point-by-point IFE correction for the synchronous spectra, these observations are treated as qualitative and are not used as hard evidence for a specific conformational change.

**Figure 5:**
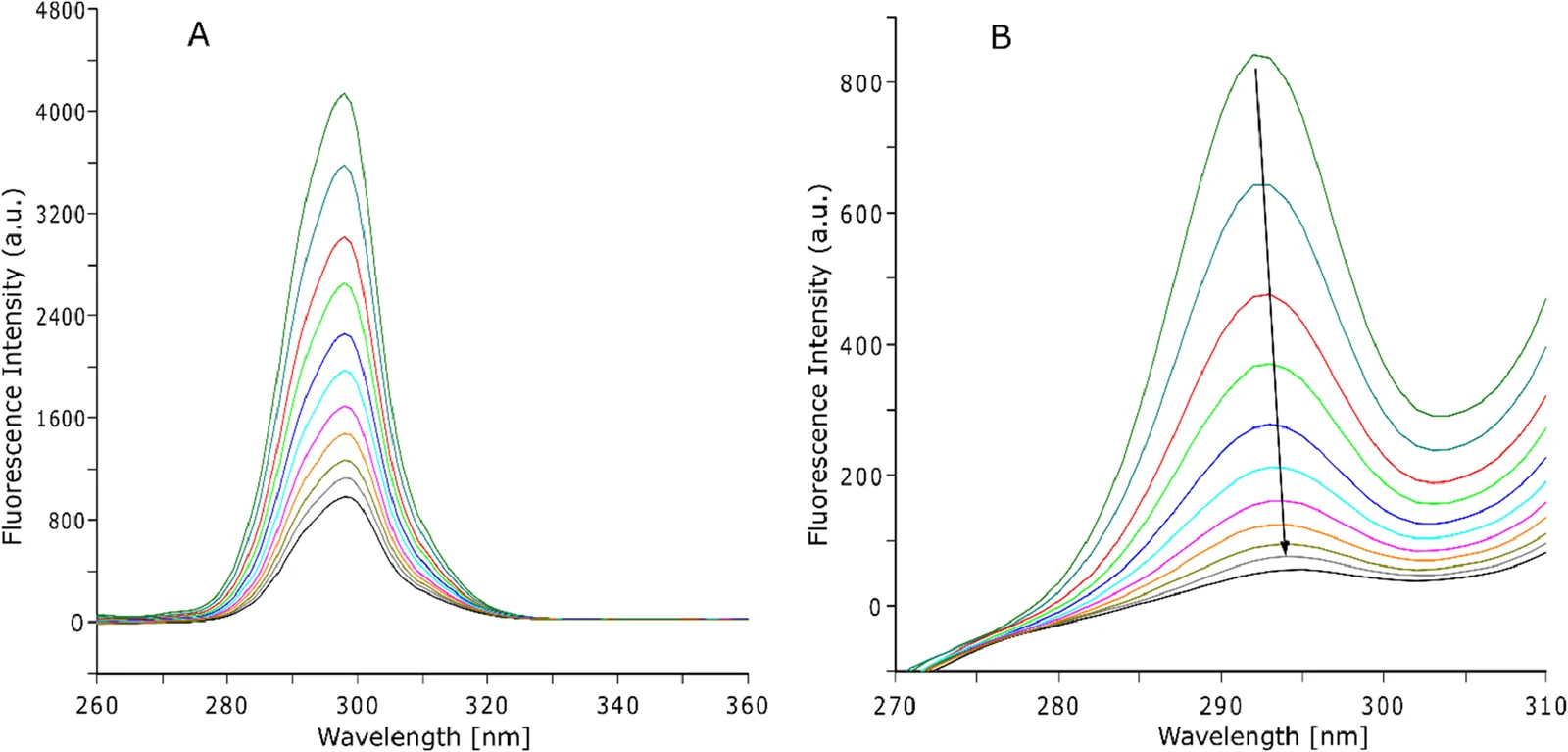
Synchronous fluorescence spectra of the HSA–LASMI complex at varying ligand concentrations: (A) Δλ = 15 nm showing tyrosine emission; (B) Δλ = 60 nm showing tryptophan emission with a slight red shift.

### 3.5 Three-Dimensional (3D EEM) Fluorescence Spectroscopy

Three-dimensional excitation-emission matrix (3D EEM) fluorescence spectroscopy provides a powerful analytical tool for visualizing global topological and conformational transformations within protein structures. As illustrated in Figure 6A, the EEM of pure albumin exhibits distinctive topographic features, including the diagonal line of Rayleigh scattering (λ_ex_ = λ_em_; a) and a primary emission peak (Peak I) centered at λ_ex_ ∼ 280 nm and λ_em_ ∼ 339 nm. This signal originates from π→π* electronic transitions within the aromatic fluorophores, mainly tryptophan and tyrosine, and is sensitive to fluctuations in its microenvironment. A secondary, less intense signal is also seen at shorter excitation wavelengths (λ_ex_ ∼ 230; Peak II). This band is often assigned to the fluorescence of the polypeptide backbone;^52^ however, as convincingly demonstrated by Bortolotti et al.,^53^ the peptide bond does not give rise to appreciable emission at ∼340 nm. Instead, in accordance with the Kasha-Vavilov rule, the coincidence of the emission maxima of Peak I and Peak II indicates that both bands originate from the same aromatic fluorophores, with Peak II corresponding to their excitation via higher-energy π→π* transitions in the far-UV region. The intensity of this band thus remains sensitive to the microenvironment of the aromatic residues and can still serve as a reporter of ligand-induced perturbations of the protein structure.

**Figure 6:**
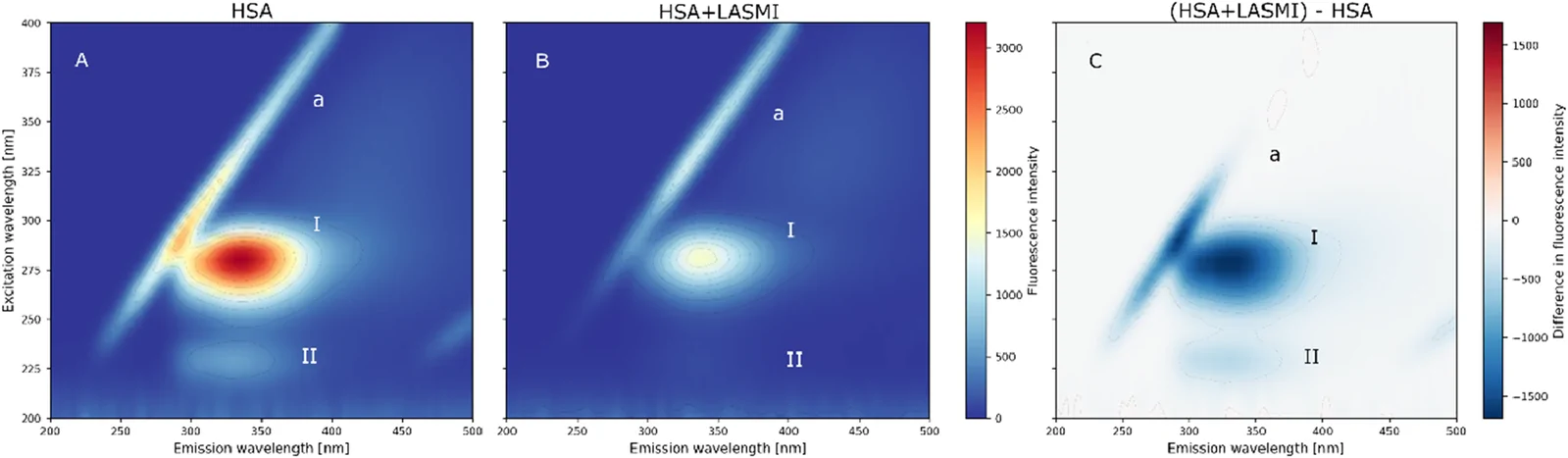
Three-dimensional excitation-emission matrix (3D EEM) fluorescence spectra of (A) native albumin and (B) its complex with 96 μM LASMI. Panel (C) shows the difference contour map, illustrating the direct quenching of Peak I and Peak II upon ligand binding.

The introduction of LASMI into the albumin solution induced changes across the spectrum (Figure 6B), which are quantitatively captured in the differential contour maps (Figure 6C). Derived by mathematically subtracting the EEM of pure HSA from those of the protein-ligand complexes, these maps shows the spatial regions with maximum intensity loss. Notably, the highest intensity deficyt (dark blue) concentrates at the center of Peak I, confirming the quenching of aromatic residues resulting from interaction with the ligand.

Furthermore, evaluation of the raw 3D EEM coordinates reveals the bathochromic shift (from 339 nm toward 343 nm) of the Peak I, also observed in 2D steady-state titrations. This red shift suggests a change in the polarity of the microenvironment of the Trp/Tyr residues, accompanied by a concurrent decrease in the emission of the aromatic fluorophores, manifested as the quenching of both Peak I and Peak II. It is highly plausible that LASMI penetrates the binding cavity and positions its polar moieties in close proximity to tryptophan; together with the hydrogen bonding network. Consequently, the conformational freedom of the fluorophores to undergo electronic transitions lessens, leading to the reduction in emission.^54,55^

### 3.6 Thermodynamic Parameters and Nature of Binding Forces

The specific type of non-covalent interactions driving protein-ligand complexation depends on the relative interplay of hydrogen bonding, van der Waals forces, electrostatic attractions, and hydrophobic effects. Elucidating the predominant binding mechanism requires evaluating the standard enthalpy change (Δ*H*) and entropy change (Δ*S*) of the system. These parameters were derived from the slope and intercept of the linear regression plot of the natural logarithm of the association constant against the reciprocal of absolute temperature, in accordance with the van’t Hoff equation:

6 $$\ln K_{a}=-\frac{\Delta H}{\mathrm{RT}}+\frac{\Delta S}{R}$$

where *R* denotes the universal gas constant and *T* represents the temperature in kelvins. The standard Gibbs free energy change (Δ*G*) at each respective temperature was subsequently calculated using the relationship:

7 ΔG=ΔH−TΔS

As shown in Figure 7 and Table 2, linear regression revealed an exothermic reaction with Δ*H* = −45.75 kJ/mol and a negative entropy change (Δ*S* = −80.44 J/(mol·K)). According to classical thermodynamic guidelines,^56,57^ this specific signature (Δ*H* < 0, Δ*S* < 0) confirms that hydrogen bonding and van der Waals interactions act as the primary forces driving complex formation. Negative Δ*G* values (−21.76, −20.96, and −20.16 kJ/mol at 298, 308, and 318 K) confirm binding spontaneity. Furthermore, as expected for an enthalpy-driven process accompanied by a reduction in conformational freedom, this spontaneity decreases slightly at elevated temperatures.

**Figure 7:**
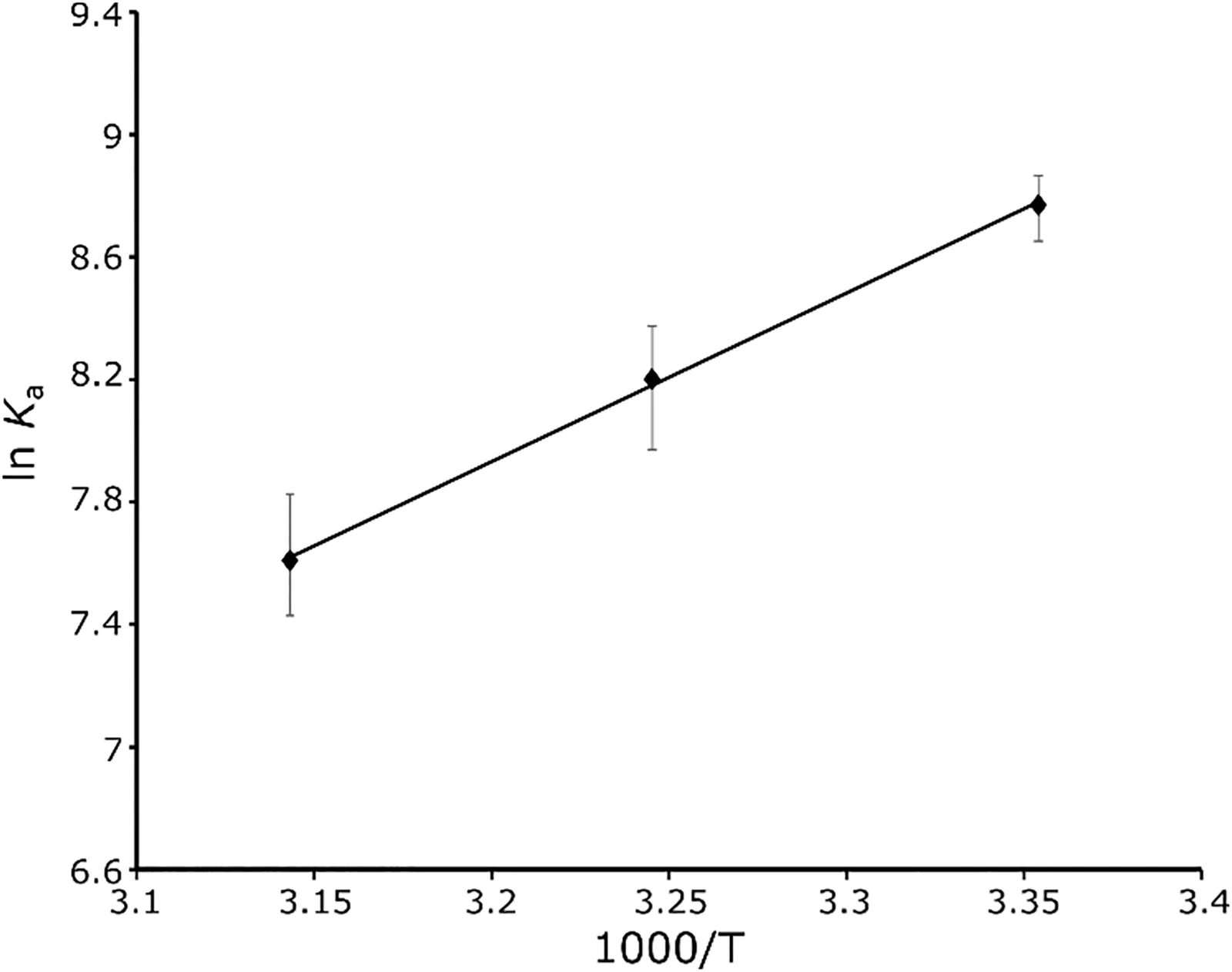
van’t Hoff linear regression plot for the HSA-LASMI binding interaction across three different temperatures (*R* ^2^ = 0.9991).

**Table 2:** Thermodynamic Parameters of the HSA-LASMI Complex at 298, 308, and 318 K

*T* [K]	Δ*H* [kJ mol^–1^]	Δ*S* [J mol^–1^ K^–1^]	Δ*G* [kJ mol^–1^]
298	–45.75 ± 6.77	–80.44 ± 22.50	–21.76 ± 0.36
308			–20.96 ± 0.39
318			–20.16 ± 0.53

### 3.7 Competitive Binding Site Analysis

Human serum albumin has two main binding sites: Sudlow Site I (subdomain IIA), Sudlow Site II (subdomain IIIA), and the recently identified binding Site III (subdomain IB).^18,58^ To identify the binding site of the HSA-LASMI complex, the competitive displacement method was used. Warfarin (Site I), ibuprofen (Site II), and lidocaine (Site III) were used as markers.

The fluorescence titrations were repeated in the presence of equimolar site markers. HSA and each marker were used at 4 μM, and LASMI was added at 16–96 μM. For each sample, absorbance was measured at 280 and 340 nm, and the fluorescence data were corrected for the inner filter effect before calculation of the association constant (Figure 8). The apparent *K*_a_ for LASMI binding to HSA at 298 K in control samples was (6.398 ± 0.727) × 10^3^ M^–1^ (consistent with the independently determined value reported in Table 1). In the presence of warfarin, *K*_a_ decreased to (1.937 ± 0.739) × 10^3^ M^–1^. This decrease of about 70% shows that prior occupation of the warfarin-sensitive region strongly weakens LASMI binding to HSA. In contrast, ibuprofen and lidocaine had no comparable effect: *K*_a_ was (6.206 ± 0.911) × 10^3^ M^–1^ with ibuprofen and (6.684 ± 0.920) × 10^3^ M^–1^ with lidocaine, both within the variation of the control (Table 3). The selective effect of warfarin therefore points to the warfarin-sensitive Sudlow Site I as the preferred binding region for LASMI. These results should be read as competition data, not as direct proof of complete displacement or of binding to only one site. The assay measures how each marker changes the fluorescence-derived association constant. Still, the strong effect of warfarin, combined with the minimal effect from ibuprofen and lidocaine, supports LASMI binding at Site I.

**Figure 8:**
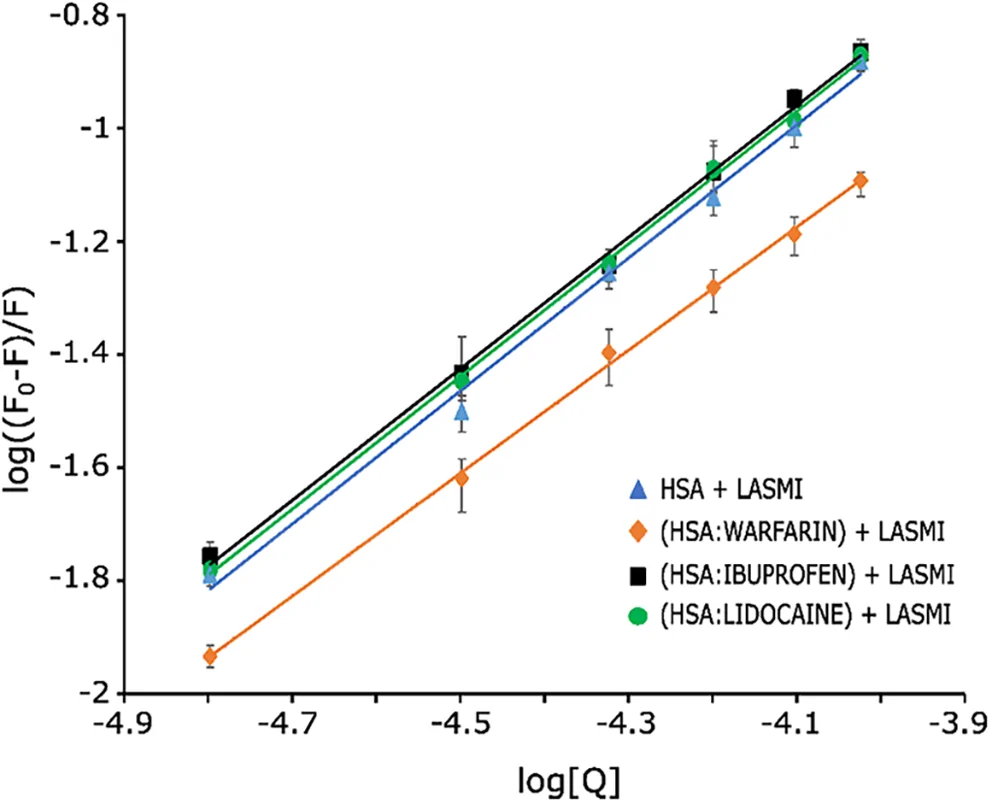
Double-logarithmic plots for LASMI association with HSA in the absence and presence of equimolar site-marker ligands at 298 K. HSA and each marker were present at 4 μM; LASMI concentrations ranged from 16 to 96 μM (*n* = 3).

**Table 3:** Association Constants for LASMI Binding to HSA in the Absence and Presence of Site-Marker Ligands at 298 K (*n* = 3)

system	*K*_a_ (× 10^3^ M^–1^), mean ± SD	*R*
HSA + LASMI control	6.398 ± 0.727	0.9959
HSA:warfarin + LASMI	1.937 ± 0.739	0.9979
HSA:ibuprofen + LASMI	6.206 ± 0.911	0.9955
HSA:lidocaine + LASMI	6.684 ± 0.920	0.9976

### 3.8 Circular Dichroism Analysis

Circular dichroism analysis is based on measuring differences in the absorption of left- and right-circularly polarized light. These differences appear in compounds that have a chiral chromophore or, more commonly, when a chromophore is near a chiral atom, as in proteins. The far-UV CD spectrum of proteins contains information about peptide bond electronic transitions: *n*→π* (around 215–225 nm) and π→π* (190–215 nm), as well as aromatic side chains (260–300 nm).^59^

The far-UV CD spectra of HSA showed the two characteristic negative bands near 208 and 222 nm, which come mainly from the α-helical structure (Figure 9). Adding LASMI caused a small, concentration-dependent decrease in the intensity of both bands, without any clear shift of their positions. Analysis of the signal at 222 nm showed that [θ]_222_ changed from −18445.9 ± 13.9 to −17710.1 ± 44.9° cm^2^ dmol^–1^ when the LASMI concentration was increased from 0 to 96 μM. Using the Chen-Yang equation, the apparent α-helical content decreased from 53.16 ± 0.05% for free HSA to 50.73 ± 0.15% at 96 μM LASMI (Table 4). This corresponds to a small decrease of about 2.4 percentage points in the α-helical content under in vitro conditions.

**Table 4:** Mean Residue Ellipticity at 222 nm and Apparent α-Helical Content of HSA in the Presence of Increasing LASMI Concentrations (*n* = 3)

LASMI [μM]	[θ]_222_, mean ± SD [deg cm^2^ dmol^–1^]	α-helix, mean ± SD [%]
0	–18445.9 ± 13.9	53.16 ± 0.05
32	–18208.0 ± 20.2	52.37 ± 0.07
64	–17967.2 ± 37.4	51.58 ± 0.12
96	–17710.1 ± 44.9	50.73 ± 0.15

**Figure 9:**
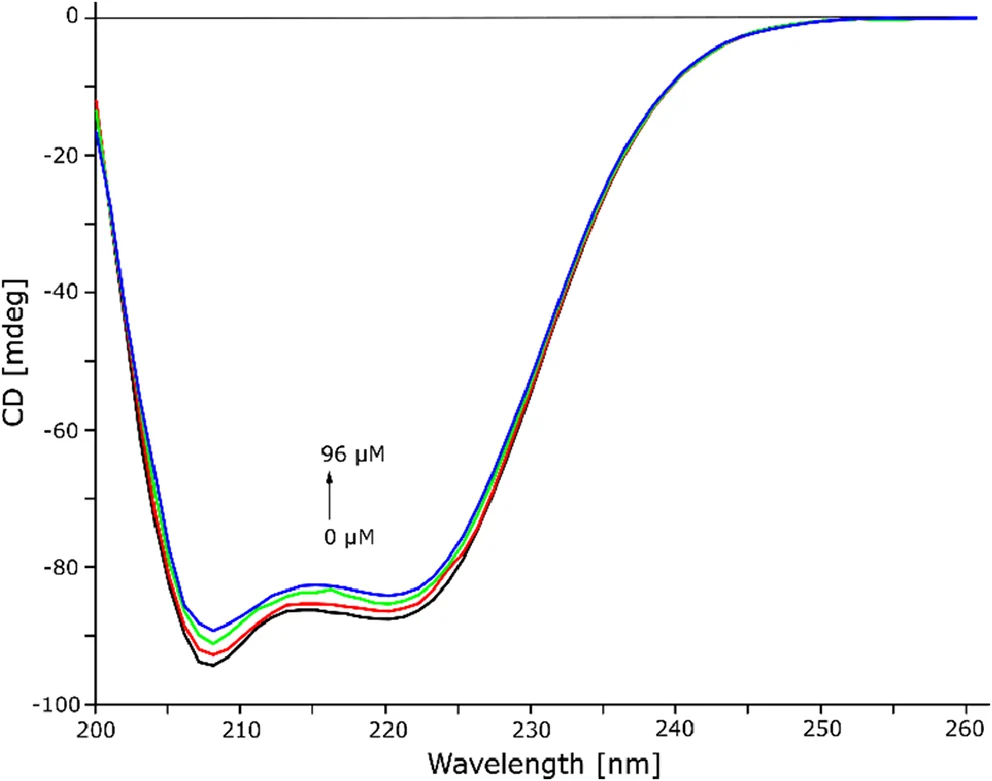
Far-UV CD spectra of pure HSA before and after the addition of 32, 64, and 96 μM lasmiditan (PBS, pH 7.4, 25 °C).

The CD results should be considered together with the MD data. Far-UV CD reflects the average conformation of the whole protein, whereas the MD simulations show reduced flexibility mainly in residues ∼200–300, which covers subdomain IIA and the modeled LASMI binding site. Thus, a small decrease in the global α-helical CD signal can exist at the same time as local stabilization of the binding pocket. These results do not indicate major unfolding of HSA; rather, they show that LASMI binding causes a small but reproducible shift in the conformational states of the protein under the experimental conditions.

### 3.9 FTIR Spectroscopy

Changes in the HSA polypeptide backbone caused by interaction with LASMI were evaluated using infrared spectroscopy. To eliminate water interference, measurements were performed in the solid state using KBr pellets after evaporating the samples under a nitrogen stream. The spectra of pure albumin and the complex were analyzed after subtracting the spectrum of pure LASMI. In the amide I band region (1700–1600 cm^–1^), no changes in peak positions were observed (Figure 10). However, in the amide II band region (1600–1500 cm^–1^), a clear shift of the peak toward higher wavenumbers is visible. The amide II band corresponds to N–H bending and C–N stretching vibrations.^60^ A shift of about 11 cm^–1^ toward higher energies may indicate hydrogen bond formation with the ligand. This stiffens the protein system, requiring more energy to trigger vibrations.

**Figure 10:**
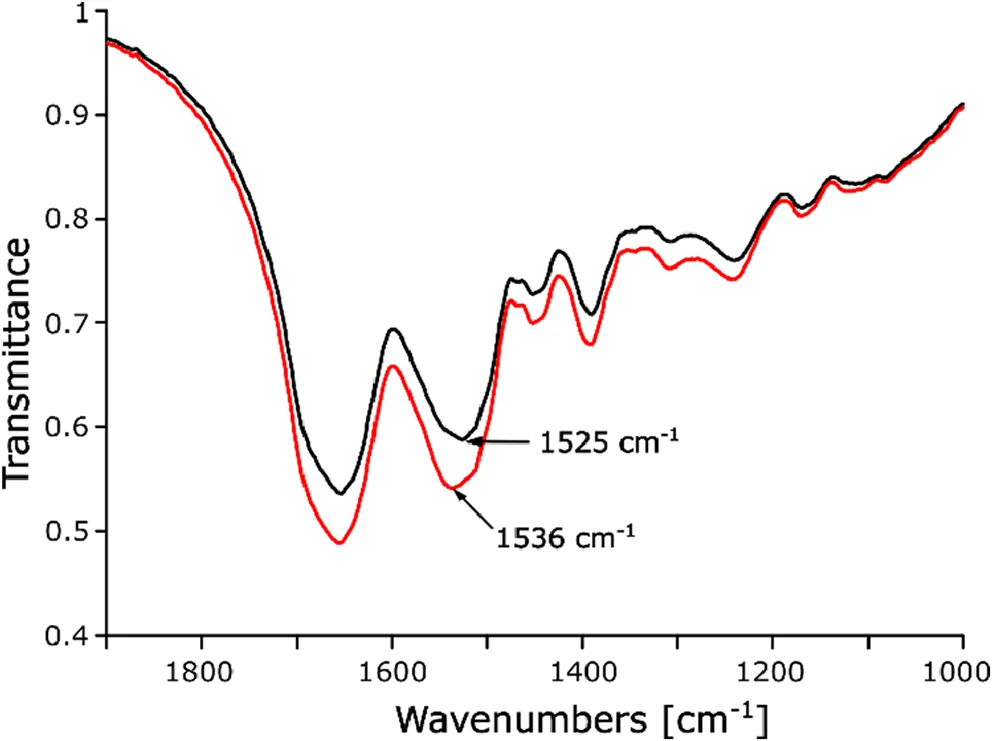
FTIR spectra of free HSA (black line) and the HSA-LASMI complex after subtracting the spectrum of pure LASMI (red line).

### 3.10 Molecular Docking

To check the reproducibility and site preference of LASMI docking, five independent blind-docking runs were performed with HSA structure 1AO6, using a 100 × 100 × 100 Å search box centered on the protein. In each run, at least two of the top-ranked LASMI poses were found in Sudlow Site I (subdomain IIA). The remaining poses were located in the Site III region or on the protein surface. Site-specific docking was then performed separately for Site I, Site II, and Site III, using a 24 × 36 × 24 Å search box. Across five independent runs (Table 5), LASMI in Site I received the best Vina scores (−9.39 ± 0.02 kcal mol^–1^), compared with Site II (−8.17 ± 0.14 kcal mol^–1^) and Site III (−8.40 ± 0.06 kcal mol^–1^). Site I was therefore consistently ranked as the most favorable of the three binding sites. The docking protocol was validated with the warfarin-bound HSA structure 2BXD. After removing crystallographic warfarin, redocking into Site I recovered the known binding mode with a heavy-atom RMSD of 1.4 ± 0.4 Å. This indicates that the docking protocol reproduced the crystallographic Site I binding mode of warfarin with good accuracy. When LASMI was docked into 2BXD with warfarin kept as a rigid part of the receptor, the best LASMI poses were shifted from the interior of Site I to the protein surface, and the Vina score dropped to −6.69 ± 0.06 kcal mol^–1^. This result agrees with the experimental finding that warfarin reduces the apparent binding constant of LASMI.

**Table 5:** Summary of Independent Molecular Docking Calculations for LASMI and Warfarin with Human Serum Albumin^a^

ligand	receptor	binding region	warfarin in receptor	top-ranked Vina score, kcal/mol
LASMI	1AO6	blind docking	no	–8.96 ± 0.08
LASMI	1AO6	subdomain IIA	no	–9.39 ± 0.02
LASMI	1AO6	subdomain IIIA	no	–8.17 ± 0.14
LASMI	1AO6	subdomain IB	no	–8.40 ± 0.06
LASMI	2BXD	blind docking	no	–9.18 ± 0.25
LASMI	2BXD	subdomain IIA	no	–8.72 ± 0.03
LASMI	2BXD	subdomain IIA	yes	–6.69 ± 0.06
Warfarin	2BXD	blind docking	no	–8.65 ± 0.33
Warfarin	2BXD	subdomain IIA, redocking	no	–9.35 ± 0.01

a Vina scores are reported as relative, model-dependent ranking values and are not direct estimates of experimental binding free energies. Values are mean ± SD of the first-ranked poses from five independent docking runs.

Analysis of the top-ranked docking pose in subdomain IIA suggested that LASMI is stabilized by a network of non-covalent interactions. The two-dimensional interaction map (Figure 11A) showed several hydrophobic and van der Waals contacts with residues lining the binding pocket, including Phe206, Arg209, Ala210, Phe211, Ala213, Val216, and Lys351. Trp214 was located in close proximity to the docked ligand. The three-dimensional visualization (Figure 11B) further indicated potential polar and aromatic interactions between LASMI and residues within Sudlow site I. The docking model suggested possible short polar contacts involving the fluorinated moiety of LASMI and residues lining Site I. The aromatic ring of the ligand forms a π-cation interaction with the Lys-351 residue. Overall, docking suggested that LASMI can be accommodated within Sudlow site I through hydrophobic contacts, van der Waals interactions, and potentially polar interactions with residues lining the pocket (Figure 12).

**Figure 11:**
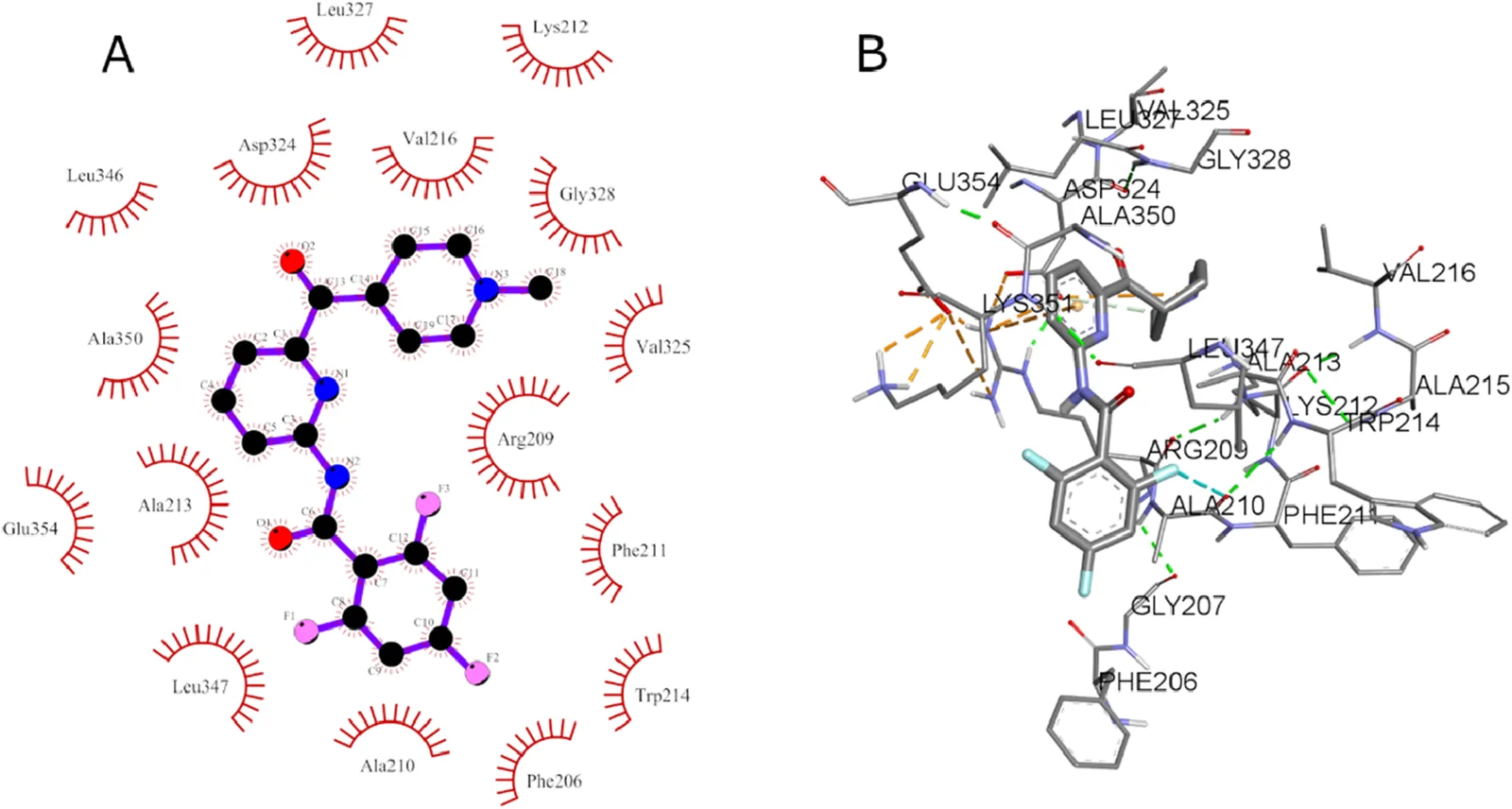
(A) 2D schematic of the LASMI binding site in HSA subdomain IIA. (B) 3D model of the complex showing the network of non-covalent interactions.

**Figure 12:**
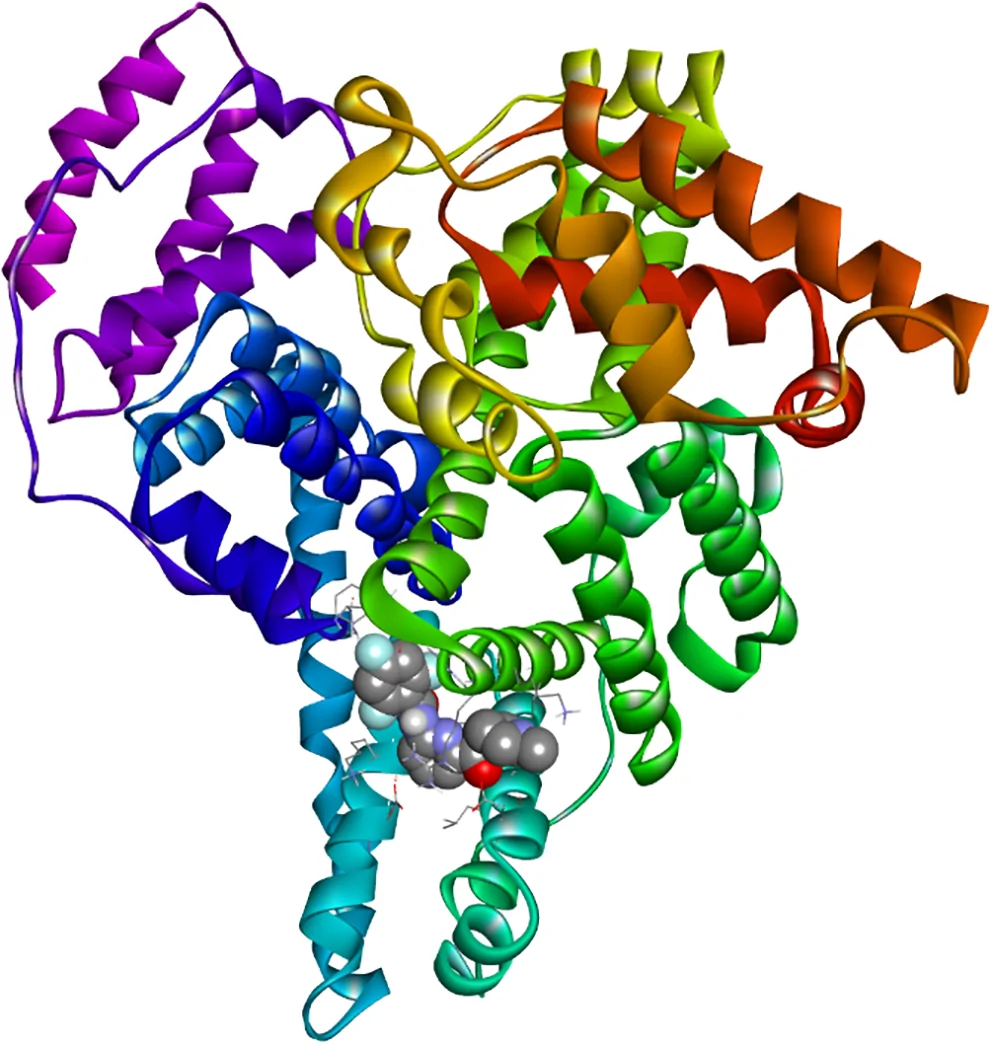
3D molecular representation of lasmiditan docked inside the primary binding site of human serum albumin.

### 3.11 Molecular Dynamics

The conformational stability of the HSA-LASMI complex relative to native albumin was verified in 100 ns molecular dynamics simulations. Three independent replicates (Runs I–III) were performed for the protein-ligand system to check simulation reproducibility.

The root-mean-square deviation (RMSD) of the backbone atoms differs between the systems (Figure 13A). For pure albumin, equilibrium is reached after about 50 ns, with a deviation around 0.30–0.34 nm. In the presence of LASMI, the RMSD curves for all runs are noticeably lower, reaching a plateau in the 0.22–0.26 nm range. A decrease in the RMSD parameter may indicate a stiffening of the protein structure upon ligand binding, which is also confirmed by experimental data.

**Figure 13:**
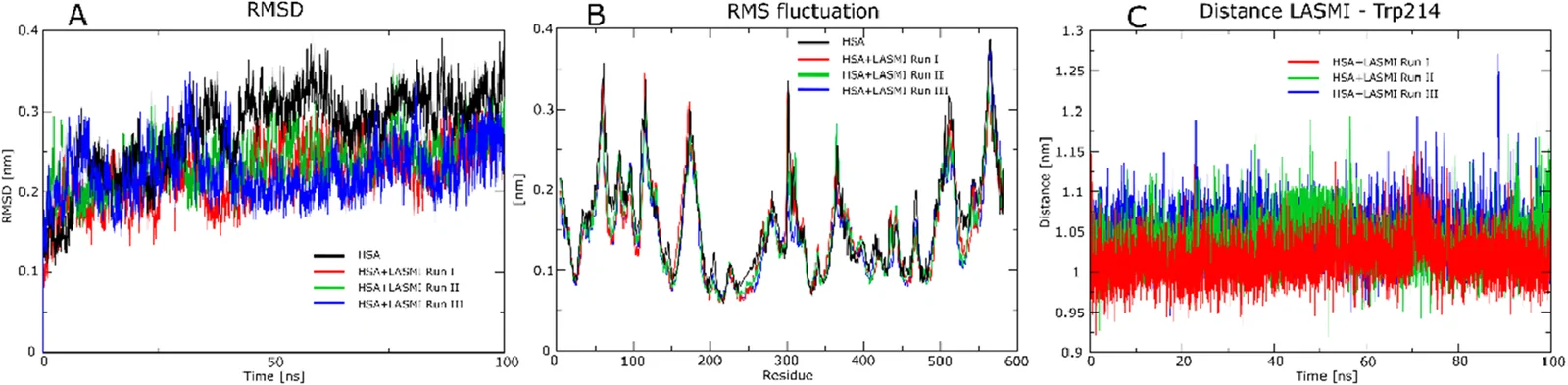
Molecular dynamics simulation analysis of free HSA and the HSA-LASMI complex over 100 ns (Runs I–III): (A) root-mean-square deviation (RMSD) of backbone atoms; (B) root-mean-square fluctuation (RMSF) of individual amino acid residues; (C) distance between the centers of mass (COM) of LASMI and residue Trp-214.

Analysis of the root-mean-square fluctuation (RMSF) of individual residues suggests that the greatest drop in polypeptide chain flexibility upon complex formation occurs between residues 200 and 300 (Figure 13B). This is noticeable for all simulation runs. This region corresponds to subdomain IIA. Trajectory analysis also showed that in each run, the number of hydrogen bonds remained at an average of 2, with rare fluctuations ranging from 0 to 4.

The distance between the centers of mass of LASMI and Trp214 remained within 1.00–1.10 nm throughout the simulations. In all three runs, LASMI remained within the binding pocket during the 100 ns production period (Figure 13C). In Run III, a transient increase in the distance to approximately 1.25 nm was observed at around 88 ns; however, the ligand subsequently returned to its previous position within the pocket. The persistent LASMI-Trp214 distance of approximately 1.05 nm indicates that the ligand remained in close proximity to the intrinsic fluorophore, which is consistent with the experimentally observed perturbation of the Trp microenvironment and the bathochromic shift in the fluorescence spectrum.^61^

The binding free energy (Δ*G*_bind_) of the HSA-LASMI complex was estimated using the gmx_MMPBSA tool based on molecular dynamics trajectories. The MM/PBSA results obtained for the three independent simulation runs (Runs I–III) are summarized in Table 6. The mean total binding free energy (Δ*G*_bind_) was −21.59 ± 0.84 kcal/mol, indicating a favorable energetic profile for LASMI binding within the HSA pocket. Overall, MM/PBSA calculations supported the energetic stability of the modeled HSA-LASMI complex.

**Table 6:** Interaction Energy Components Calculated with gmx_MMPBSA Method

energy component [kcal/mol]	Run I	Run II	Run III	average
van der Waals contribution	–37.4	–38.85	–38.62	–38.29 ± 0.78
electrostatic contribution	–132.81	–131.98	–132.45	–132.41 ± 0.42
polar contribution to the solvation free energy	151.75	153.49	154.36	153.2 ± 1.32
non-polar contribution to the solvation free energy	–4.03	–4.10	–4.12	–4.08 ± 0.05
Δ*G*_gas_	–170.21	–170.83	–171.07	–170.70 ± 0.44
Δ*G*_solv_	147.72	149.39	150.24	149.12 ± 1.28
Δ*G*_total_	–22.50	–21.44	–20.84	–21.59 ± 0.84

The experimental binding free energy obtained from the van’t Hoff analysis of the fluorescence data at 298 K (Δ*G°*exp = −21.76 kJ/mol, i.e., about −5.20 kcal/mol) was smaller in magnitude than the MM/PBSA value calculated for the modeled complex (Δ*G*_MM/PBSA_ = −21.59 ± 0.84 kcal/mol). Although both methods show that binding is favorable, the two numbers should not be compared directly. The experimental value comes from an equilibrium measurement in solution, whereas MM/PBSA is an approximate, end-point method based on a limited number of structures taken from an MD trajectory. In the present calculations, the entropy change upon binding (conformational, translational, and rotational contributions) was not included, which makes the calculated value too negative. In addition, the MM/PBSA result depends on the force field, the implicit solvent model and its dielectric parameters, the length of the MD sampling, and the chosen binding pose. For these reasons, the MM/PBSA result is treated here as qualitative support for the energetic stability of the HSA-LASMI complex, and not as a direct prediction of the experimental binding free energy.

### 3.12 Biological Relevance

The association constant measured in PBS at 298 K (*K*_a_ = 6.46 × 10^3^ M^–1^) corresponds to a dissociation constant of about 155 μM, which indicates weak-to-moderate binding of LASMI to HSA under the experimental conditions. However, this value should be considered together with the very high concentration of albumin in plasma (about 0.6 mM). Even moderate binding can therefore affect the balance between the bound and unbound fractions of LASMI. In a simple 1:1 model, an albumin concentration of 0.6 mM would predict that a large fraction of LASMI is bound; however, this estimate cannot be directly transferred to plasma. *In vivo*, albumin binding is affected by endogenous ligands, competing drugs, other plasma proteins, and the limitations of the fluorescence-based binding model. For these reasons, this result is best seen as being in line with the reported moderate plasma protein binding of LASMI (55–60%),^23,24^ rather than as a direct prediction of the bound fraction in vivo.

The pharmacokinetic relevance of this interaction is also limited by the clinical concentrations and the elimination of LASMI. Lasmiditan is given orally, reaches peak plasma concentration after about 1.8 h, and is eliminated with a half-life of about 5.7 h. At therapeutic doses, the plasma concentration of LASMI is much lower than the concentration of HSA, so LASMI is not expected to saturate albumin or to change the overall structure of circulating HSA. The small spectral changes observed in this work should therefore be regarded as evidence of a local change in the binding site, observed *in vitro*. Binding at Site I may act as a temporary reservoir for LASMI and may, in principle, lead to competition with other Site I drugs. However, given the moderate affinity and the relatively short elimination half-life of LASMI, presented data do not support a role for albumin as a long-circulating carrier for this drug. Studies in human plasma and drug–drug interaction experiments at clinically relevant concentrations would be needed to determine the *in vivo* importance of this interaction.

## 4 Conclusions

The combined biophysical and computational analyses indicate that lasmiditan (LASMI) forms a spontaneous and stable complex with human serum albumin (HSA). In competition experiments, warfarin lowered the apparent binding constant of LASMI by about 70%, while ibuprofen and lidocaine had no such effect. This indicates that LASMI binds mainly to the warfarin-sensitive Sudlow Site I of HSA. The fluorescence-quenching data are consistent with a predominantly static quenching mechanism, as supported by the temperature dependence of the Stern–Volmer constants and the calculated bimolecular quenching rate constants. Synchronous fluorescence measurements indicated perturbation of the Trp microenvironment, while molecular dynamics simulations showed that LASMI remained in close proximity to Trp214, with a ligand-Trp214 distance of approximately 1.05 nm throughout the 100 ns simulations.

Experimental thermodynamic parameters indicated that LASMI binding is thermodynamically favorable, whereas MM/PBSA calculations supported a favorable energetic profile of the modeled HSA-LASMI complex, with a mean binding free energy of −21.59 kcal/mol. Circular dichroism analysis showed a small, concentration-dependent decrease in the α-helical content of HSA (from 53.2 to 50.7% at 96 μM LASMI). This change should not be read as direct evidence of a local secondary-structure transition but, together with MD, as a small shift in HSA conformational states accompanied by reduced flexibility near the modeled Site I pocket.

Overall, these findings provide molecular-level insight into HSA-LASMI association and may be relevant to the plasma distribution of lasmiditan. Preferential binding within Sudlow site I also suggests potential competition with co-administered drugs that bind in this region. The pharmacokinetic and clinical consequences of such competition, including possible effects on the unbound fraction of LASMI and central nervous system exposure, require further investigation

## Supplementary Material



## Notes

UV–Vis absorbance data; docking grid-box centers and docking settings; and complete AutoDock Vina docking scores for independent docking runs (PDF)
