# Supplementary material for: Molecular Characterization of Lasmiditan Binding to Human Serum Albumin: Site Preference, Structural Response, and Binding Energetics
Source: ACS Omega. 2026 Sep 30;11(40):62126–38. doi: 10.1021/acsomega.6c08646 (PMC13670247; doi:10.1021/acsomega.6c08646)
Supplement: Supplementary file 1 [file ao6c08646_si_001.pdf]

Supporting Information for Publication

Molecular Characterization of Lasmiditan Binding to Human Serum Albumin: Site Preference, Structural Response, and Binding Energetics

Tadeusz W. Inglot*

* E-mail: tadeusz.inglot@umlub.edu.pl

Department of Medicinal Chemistry, Faculty of Pharmacy, Medical University of Lublin, Jaczewskiego 4, 20-090 Lublin, Poland

Contents

Table S1. Corrected mean absorbance values with standard deviations for HSA-LASMI complexes measured by UV-Vis spectroscopy (*n* = 3)

LASMI [µM]	Absorbance, mean ± SD	ΔA
0	0.216 ± 0.002	0
9.6	0.210 ± 0.011	0.006
19.2	0.206 ± 0.006	0.01
28.8	0.204 ± 0.005	0.012
38.4	0.202 ± 0.01	0.014
48	0.199 ± 0.005	0.017
57.6	0.196 ± 0.006	0.02
67.2	0.195 ± 0.009	0.021
76.8	0.192 ± 0.006	0.024
86.4	0.190 ± 0.011	0.026
96	0.187 ± 0.007	0.029

Table S2. Docking settings used for the independent docking calculations

Calculation	Receptor	Warfarin retained	Grid centre, Å, (x,y,z)	Grid size, Å (x,y,z)	Runs	Exhaustiveness
LASMI blind docking	1AO6, chain A	No	29, 31, 23	100,100,100	5	32
LASMI Site I	1AO6, chain A	No	Trp214-centered 24, 36, 33	24,36,24	5	32
LASMI Site II	1AO6, chain A	No	Tyr411-centered 13, 31, 14	24,36,24	5	32
LASMI Site III	1AO6, chain A	No	Arg186-centered 33, 23, 16	24,36,24	5	32
LASMI blind docking	2BXD, chain A	No	-0.1, -0,7, 0.1	100,100,100	5	32
LASMI Site I	2BXD, chain A	No	1, -12, -2	24,36,24	5	32
LASMI Site I	2BXD, chain A	Yes	1, -12, -2	24,36,24	5	32
Warfarin redocking	2BXD, chain A	No	1, -12, -2	24,36,24	5	32

Table S3. Complete AutoDock Vina output for five independent docking runs of LASMI and warfarin with HSA. For each output pose, the Vina score and RMSD lower-bound and upper-bound values relative to the top-ranked pose within the same run are reported

LASMI 1AO6 blind															
No	Vina score	RMSD l.b	RMSD u.b.	Vina score	RMSD l.b	RMSD u.b.	Vina score	RMSD l.b	RMSD u.b.	Vina score	RMSD l.b	RMSD u.b.	Vina score	RMSD l.b	RMSD u.b.
1	-9.0689	0.00	0.00	-8.9505	0.00	0.00	-8.8658	0.00	0.00	-9.0101	0.00	0.00	-8.9246	0.00	0.00
2	-9.0323	3.65	8.14	-8.6474	3.65	8.54	-8.5155	3.76	9.40	-8.9536	0.90	2.06	-8.9178	0.21	1.59
3	-8.8565	3.60	8.33	-8.3389	2.33	3.77	-8.3857	9.98	14.94	-8.6554	36.18	39.41	-8.6830	3.73	9.42
4	-8.7561	36.40	39.53	-8.2350	3.57	9.21	-8.2884	36.19	39.24	-8.6495	36.08	39.16	-8.4983	3.30	8.28
5	-8.7138	25.80	29.82	-8.1784	36.12	39.20	-8.2664	10.10	15.24	-8.2093	10.04	15.27	-8.4879	36.16	39.21
6	-8.6481	35.57	38.63	-8.1294	37.61	39.64	-8.2377	3.63	8.34	-8.0848	7.44	11.40	-8.3141	9.95	15.05
7	-8.5981	38.65	41.79	-7.8446	2.71	4.25	-8.1685	3.49	9.04	-7.9454	2.49	3.97	-8.1319	3.67	8.45
8	-8.1129	39.30	42.49	-7.8257	36.38	39.08	-8.0197	7.73	11.66	-7.8894	1.88	2.86	-7.9726	1.77	2.34
9	-8.0981	34.66	38.15	-7.6782	4.42	9.98	-7.9877	2.53	4.09	-7.8395	3.76	8.40	-7.8001	2.45	4.09

LASMI 1AO6 Site I															
No	Vina score	RMSD l.b	RMSD u.b.	Vina score	RMSD l.b	RMSD u.b.	Vina score	RMSD l.b	RMSD u.b.	Vina score	RMSD l.b	RMSD u.b.	Vina score	RMSD l.b	RMSD u.b.
1	-9.4117	0.00	0.00	-9.3662	0.00	0.00	-9.4015	0.00	0.00	-9.3974	0.00	0.00	-9.3628	0.00	0.00
2	-9.3195	0.20	1.59	-9.2817	0.16	1.58	-8.8261	0.34	1.76	-8.9609	0.32	2.76	-8.8412	0.70	0.24
3	-8.8279	3.78	8.57	-8.8497	3.72	8.23	-8.7890	3.77	8.69	-8.8704	3.69	8.22	-8.7861	3.58	7.96
4	-8.8153	3.71	8.27	-8.8115	3.80	8.63	-8.7843	3.73	8.26	-8.7986	3.69	8.23	-8.6920	3.81	8.94
5	-8.7621	3.68	8.11	-8.7501	3.48	8.04	-8.6387	3.74	8.97	-8.7103	3.52	7.95	-8.5954	3.71	8.29
6	-8.4445	3.42	8.04	-8.5901	3.67	8.16	-8.2997	3.64	8.51	-8.4525	3.65	8.01	-8.4034	3.67	8.39
7	-8.3312	3.68	8.97	-8.3434	3.65	8.45	-8.2575	3.74	9.02	-8.3639	3.68	8.42	-8.2690	3.57	8.33
8	-8.2326	3.55	8.38	-8.3043	3.67	8.40	-8.1373	3.67	9.31	-8.1776	3.66	9.28	-8.1593	3.69	8.91
9	-8.0522	3.61	9.29	-8.1084	3.71	9.28	-8.1258	3.84	8.87	-8.1250	3.89	8.81	-8.1000	3.56	9.26

LASMI 1AO6 Site II															
No	Vina score	RMSD l.b	RMSD u.b.	Vina score	RMSD l.b	RMSD u.b.	Vina score	RMSD l.b	RMSD u.b.	Vina score	RMSD l.b	RMSD u.b.	Vina score	RMSD l.b	RMSD u.b.
1	-8.1114	0.00	0.00	-8.15	0.00	0.00	-8.0221	0.00	0.00	-8.1868	0.00	0.00	-8.3862	0.00	0.00
2	-7.9963	0.83	1.14	-7.92	1.89	2.65	-7.7863	3.01	3.78	-7.8255	1.68	2.70	-7.8406	2.44	3.10
3	-7.9635	1.97	2.91	-7.86	2.54	3.59	-7.7756	0.85	1.85	-7.7544	3.04	4.03	-7.7967	1.69	2.64
4	-7.8497	2.09	2.46	-7.72	1.93	2.84	-7.7693	2.59	3.00	-7.7184	1.97	3.06	-7.6776	2.02	3.12
5	-7.8400	2.54	3.70	-7.70	2.72	3.59	-7.7411	1.69	2.73	-7.5824	1.37	2.37	-7.6040	1.55	2.83
6	-7.8227	2.51	3.31	-7.57	2.02	4.36	-7.6333	1.97	2.90	-7.5708	2.03	4.39	-7.4451	2.36	3.38
7	-7.7579	1.90	2.69	-7.56	1.51	2.79	-7.5091	1.75	2.23	-7.4871	1.94	3.89	-7.4409	2.04	4.24
8	-7.5265	2.14	4.80	-7.49	2.18	4.83	-7.4922	1.96	4.33	-7.3864	2.66	5.17	-7.3739	2.57	4.88
9	-7.3023	2.50	3.22	-7.19	2.44	3.27	-7.1565	2.06	2.82	-7.2122	2.07	2.82	-7.1709	2.40	3.34

LASMI 1AO6 Site III															
No	Vina score	RMSD l.b	RMSD u.b.	Vina score	RMSD l.b	RMSD u.b.	Vina score	RMSD l.b	RMSD u.b.	Vina score	RMSD l.b	RMSD u.b.	Vina score	RMSD l.b	RMSD u.b.
1	-8.3537	0.00	0.00	-8.3412	0.00	0.00	-8.3732	0.00	0.00	-8.4646	0.00	0.00	-8.4757	0.00	0.00
2	-8.2906	2.12	3.44	-8.2272	2.21	3.62	-8.3283	0.24	1.59	-8.4325	2.21	3.01	-8.4444	2.22	3.45
3	-8.1422	2.96	3.89	-8.0855	1.58	2.47	-7.9224	1.66	2.10	-8.4073	2.10	3.44	-8.4132	0.57	1.68
4	-8.0205	2.89	4.13	-7.9801	1.65	2.05	-7.8973	3.96	8.91	-8.1846	2.94	4.14	-8.2682	1.56	2.45
5	-7.7707	4.58	8.81	-7.6830	5.81	10.80	-7.6114	1.67	2.64	-8.1481	2.87	3.78	-7.9676	3.89	8.90
6	-7.4717	4.33	8.80	-7.5923	3.91	8.91	-7.5614	6.57	11.09	-8.0308	3.29	4.58	-7.9538	1.55	1.89
7	-7.4676	8.92	11.99	-7.4024	6.48	11.03	-7.4202	3.71	8.90	-7.8398	4.69	8.89	-7.4805	3.66	8.80
8	-7.4663	8.02	10.90	-7.2629	6.71	10.59	-7.2265	7.42	11.85	-7.5774	8.72	11.53	-7.4574	6.57	11.14
9	-7.2011	8.61	11.57	-7.1425	7.32	11.80	-7.0984	7.29	11.79	-7.5096	4.35	8.74	-7.3847	7.30	11.76

LASMI 2BXD Blind															
No	Vina score	RMSD l.b	RMSD u.b.	Vina score	RMSD l.b	RMSD u.b.	Vina score	RMSD l.b	RMSD u.b.	Vina score	RMSD l.b	RMSD u.b.	Vina score	RMSD l.b	RMSD u.b.
1	-9.3451	0.00	0.00	-8.7430	0.00	0.00	-9.2667	0.00	0.00	-9.1863	0.00	0.00	-9.3403	0.00	0.00
2	-9.1364	1.40	2.13	-8.6940	1.37	2.09	-9.2261	1.29	2.13	-8.8640	3.82	8.40	-8.7110	2.38	3.22
3	-8.8283	3.82	8.36	-8.4387	15.05	17.25	-8.8211	3.88	8.40	-8.7308	1.30	2.16	-8.5135	3.95	8.34
4	-8.6383	13.69	14.98	-8.3689	24.38	27.93	-8.2477	3.77	8.22	-8.5609	36.43	38.60	-8.5069	3.94	8.37
5	-8.5769	22.85	26.88	-8.3479	39.80	41.24	-8.1981	4.62	7.79	-8.5532	38.58	39.96	-8.3808	38.49	39.91
6	-8.4229	36.98	38.88	-8.2852	37.77	39.77	-8.1771	22.69	26.68	-8.4256	22.96	26.94	-8.3512	22.80	26.92
7	-8.3776	36.91	38.84	-8.2228	38.90	39.87	-8.0160	36.50	38.59	-8.1358	4.56	7.61	-8.3236	36.37	38.56
8	-8.1494	3.79	8.38	-8.1749	37.09	39.78	-7.7911	36.50	38.50	-7.9215	37.78	39.73	-8.2593	35.82	38.62
9	-8.0635	23.11	27.06	-8.0337	4.47	10.50	-7.7614	38.65	39.77	-7.7794	38.66	39.80	-8.0700	37.13	38.79

LASMI 2BXD Site I															
No	Vina score	RMSD l.b	RMSD u.b.	Vina score	RMSD l.b	RMSD u.b.	Vina score	RMSD l.b	RMSD u.b.	Vina score	RMSD l.b	RMSD u.b.	Vina score	RMSD l.b	RMSD u.b.
1	-8.7115	0.00	0.00	-8.6901	0.00	0.00	-8.7069	0.00	0.00	-8.7774	0.00	0.00	-8.7284	0.00	0.00
2	-8.6429	1.66	2.46	-8.5260	1.64	2.36	-8.5649	1.75	2.32	-8.6341	1.59	2.27	-8.6756	1.67	2.42
3	-8.5739	1.90	2.74	-8.4723	1.90	2.73	-8.5544	1.96	2.76	-8.5190	1.70	2.01	-8.5362	1.75	2.03
4	-8.4422	3.75	8.26	-8.4136	3.75	8.28	-8.4108	3.42	8.24	-8.5179	1.97	2.86	-8.5092	3.74	8.21
5	-8.3500	3.44	8.54	-8.3257	3.40	8.55	-8.3944	3.38	8.21	-8.4630	3.75	8.22	-8.4283	3.43	8.21
6	-8.3096	3.88	8.93	-8.2409	4.60	7.45	-8.2313	3.65	8.32	-8.3912	3.90	8.87	-8.3707	3.40	8.18
7	-8.1539	1.57	2.50	-8.1995	3.88	8.94	-8.2294	3.95	8.56	-8.3804	3.40	8.47	-8.3150	1.79	2.32
8	-7.6040	2.86	4.98	-8.1764	1.52	2.46	-8.1799	3.96	8.69	-8.3406	3.65	8.16	-8.2450	3.98	8.67
9	-7.4588	4.15	7.21	-8.1025	4.26	8.91	0.8064	1.67	2.41	-8.2926	1.57	2.51	-8.1169	2.23	3.31

LASMI 2BXD Site I with warfarin retained															
No	Vina score	RMSD l.b	RMSD u.b.	Vina score	RMSD l.b	RMSD u.b.	Vina score	RMSD l.b	RMSD u.b.	Vina score	RMSD l.b	RMSD u.b.	Vina score	RMSD l.b	RMSD u.b.
1	-6.7418	0.00	0.00	-6.7380	0.00	0.00	-6.6237	0.00	0.00	-6.7273	0.00	0.00	-6.6176	0.00	0.00
2	-6.3449	22.50	25.41	-6.3730	3.49	8.56	-6.5767	0.28	1.60	-6.4675	22.27	25.26	-6.4100	3.43	8.51
3	-6.3275	3.53	8.57	-6.2940	1.49	2.24	-6.2331	3.53	8.58	-6.2003	3.54	8.47	-6.1236	1.47	2.46
4	-6.2532	1.51	2.26	-6.1777	3.55	8.46	-6.1952	3.54	8.47	-6.1876	1.59	2.36	-6.1007	3.45	8.36
5	-6.2249	3.55	8.45	-6.1054	3.44	8.40	-6.1294	3.53	8.45	-6.1286	1.02	1.71	-6.0927	3.54	8.46
6	-6.0821	1.50	2.52	-5.9821	2.91	4.89	-6.0385	1.56	2.31	-6.1109	3.43	8.39	-6.0883	1.53	2.28
7	-6.0009	3.41	8.38	-5.9144	1.56	2.76	-6.0125	3.43	8.38	-5.9165	2.99	5.00	-5.9574	3.44	8.47
8	-5.8620	3.00	5.54	-5.8071	2.97	5.48	-6.0075	2.95	4.92	-5.9123	1.57	2.77	-5.7550	3.44	6.00
9	-5.8115	3.42	5.90	-5.7936	3.39	5.85	-5.7013	1.64	2.68	-5.6653	3.42	6.02	-5.6454	3.41	5.18

Warfarin 2BXD Blind															
No	Vina score	RMSD l.b	RMSD u.b.	Vina score	RMSD l.b	RMSD u.b.	Vina score	RMSD l.b	RMSD u.b.	Vina score	RMSD l.b	RMSD u.b.	Vina score	RMSD l.b	RMSD u.b.
1	-8.8003	0.00	0.00	-8.2715	0.00	0.00	-8.9978	0.00	0.00	-8.8489	0.00	0.00	-8.3243	0.00	0.00
2	-8.2831	1.06	4.54	-8.1808	2.86	6.35	-8.6309	1.11	2.20	-8.3446	1.62	5.88	-8.2371	2.87	6.44
3	-8.1445	0.56	4.22	-7.7087	2.39	6.29	-8.1980	34.85	38.11	-7.9876	34.99	38.30	-8.2209	2.27	5.93
4	-7.8326	2.34	4.63	-7.3685	3.12	4.69	-8.1943	1.57	4.52	-7.9290	11.14	14.90	-7.9315	1.88	5.70
5	-7.6117	2.51	4.56	-6.9312	7.80	1.06	-8.0847	1.60	3.45	-7.8961	1.86	3.58	-7.5293	1.49	3.61
6	-7.5133	6.62	8.80	-6.8012	6.30	9.58	-7.9956	2.15	3.54	-7.8823	1.68	3.92	-7.4400	1.83	5.32
7	-7.4172	6.36	9.35	-6.3983	7.72	1.23	-7.2922	38.09	40.10	-7.7488	2.14	6.27	-7.3501	34.63	37.13
8	-7.3901	0.99	4.46	-6.3444	6.00	8.95	-7.1170	3.57	8.82	-7.5907	2.30	6.68	-7.1514	2.58	4.67
9	-7.1137	4.58	8.62	-6.3396	6.69	9.83	-7.0526	22.35	25.67	-7.3610	3.62	8.56	-7.1297	2.99	6.81

Warfarin 2BXD Site I															
No	Vina score	RMSD l.b	RMSD u.b.	Vina score	RMSD l.b	RMSD u.b.	Vina score	RMSD l.b	RMSD u.b.	Vina score	RMSD l.b	RMSD u.b.	Vina score	RMSD l.b	RMSD u.b.
1	-9.3550	0.00	0.00	-9.3609	0.00	0.00	-9.3493	0.00	0.00	-9.3251	0.00	0.00	-9.3434	0.00	0.00
2	-9.0828	1.94	5.39	-9.1188	1.97	5.39	-9.1577	1.96	5.37	-9.0719	1.90	5.39	-9.0796	1.93	5.38
3	-8.9010	1.09	2.44	-8.7927	1.45	3.83	-8.8473	2.05	5.11	-8.8272	2.05	5.08	-8.7724	2.06	5.11
4	-8.8255	1.43	3.81	-8.7714	2.10	5.14	-8.7743	0.92	2.23	-8.7897	0.90	2.21	-8.7332	1.50	3.69
5	-8.8095	2.10	5.12	-8.7502	0.96	2.25	-8.7646	1.54	3.82	-8.5342	1.45	3.65	-8.7131	0.93	2.23
6	-8.7971	0.94	2.24	-8.4038	1.80	5.93	-8.4883	1.78	5.96	-8.4234	1.77	5.94	-8.4067	1.79	5.94
7	-8.4311	1.95	5.74	-8.2933	1.89	5.83	-8.1437	2.54	6.47	-8.2274	1.92	5.78	-8.2535	1.99	5.74
8	-8.0244	1.91	5.77	-8.1057	1.84	5.77	-8.0696	1.96	5.73	-7.7640	1.44	4.26	-7.9947	1.96	5.79
9	-7.8286	1.37	4.20	-8.0844	1.33	4.01	-7.9933	1.35	4.00	-6.4704	15.71	17.76	-7.6238	1.46	4.26
